# SIRT1 regulates differentiation of mesenchymal stem cells by deacetylating β-catenin

**DOI:** 10.1002/emmm.201201606

**Published:** 2013-01-30

**Authors:** Petra Simic, Kayvan Zainabadi, Eric Bell, David B Sykes, Borja Saez, Sutada Lotinun, Roland Baron, David Scadden, Ernestina Schipani, Leonard Guarente

**Affiliations:** 1 Glenn Laboratory for the Science of Aging, Department of Biology, Massachusetts Institute of TechnologyCambridge, MA, USA; 2 Center for Regenerative Medicine, Massachusetts General HospitalBoston, MA, USA; 3 Department of Oral Medicine, Harvard School of Dental Medicine, Infection, and ImmunityBoston, MA, USA; 4 MGH-Harvard Medical School, Endocrinology UnitBoston, MA, USA

**Keywords:** bone, cartilage, fat, mesenchymal stem cells, sirtuin

## Abstract

Mesenchymal stem cells (MSCs) are multi-potent cells that can differentiate into osteoblasts, adipocytes, chondrocytes and myocytes. This potential declines with aging. We investigated whether the sirtuin SIRT1 had a function in MSCs by creating MSC specific SIRT1 knock-out (MSCKO) mice. Aged MSCKO mice (2.2 years old) showed defects in tissues derived from MSCs; *i.e.* a reduction in subcutaneous fat, cortical bone thickness and trabecular volume. Young mice showed related but less pronounced effects. MSCs isolated from MSCKO mice showed reduced differentiation towards osteoblasts and chondrocytes *in vitro*, but no difference in proliferation or apoptosis. Expression of β-catenin targets important for differentiation was reduced in MSCKO cells. Moreover, while β-catenin itself (T41A mutant resistant to cytosolic turnover) accumulated in the nuclei of wild-type MSCs, it was unable to do so in MSCKO cells. However, mutating K49R or K345R in β-catenin to mimic deacetylation restored nuclear localization and differentiation potential in MSCKO cells. We conclude that SIRT1 deacetylates β-catenin to promote its accumulation in the nucleus leading to transcription of genes for MSC differentiation.

## INTRODUCTION

Mesenchymal stem cells (MSCs) are multipotent cells that can be isolated from bone marrow, can proliferate *in vitro* with fibroblast-like morphology, and can be induced to differentiate into a variety of mesenchymal tissues, including bone, cartilage, tendon, fat, bone marrow stroma and muscle (Pittenger et al, 1999; Prockop, 1997; Uccelli et al, 2008). MSCs can most reliably be identified by the monoclonal antibody STRO1 (Loeuillet et al, 2001), which recognizes a cell surface protein on MSCs. However, MSCs are not as well characterized as other adult stem cells, such as haematopoietic stem cells (HSCs).

Osteogenic, adipogenic, chondrogenic and myogenic potential is compromised in MSCs of aged rats (Asumda & Chase, 2011). In addition, the number of MSCs with osteogenic, chondrogenic and adipocyte potential has been reported to decline in aging potentially contributing to age-related osteoporosis, osteoarthritis and loss of sub-cutaneous fat (Cartwright et al, 2007; Chang et al, 2011; D'Ippolito et al, 1999). Aging also compromises the efficacy of MSC differentiation to myocytes and regeneration of damaged myocardial tissue (Asumda & Chase, 2011; Khan et al, 2011), leading to muscle wasting and increased frailty (Goldspink, 2012). Indeed, transplantation of MSCs from young donors delays aging in mice and prolongs the life span (Shen et al, 2011). Aging also causes an inappropriate shift of MSC commitment in bone from the osteoblast to the adipocyte lineage (Moerman et al, 2004), resulting in an increase in bone marrow adipogenesis (Jiang et al, 2008).

Wnt signalling via β-catenin plays an important but complex role in the self-renewal and differentiation of MSCs (Ling et al, 2009). Wnt signaling can exert a positive effect on self-renewal in some cases (Boland et al, 2004; Cho et al, 2006), and a negative effect in others (Qiu et al, 2007). These differences may be due to different responses of MSCs to different doses of Wnt signaling (De Boer et al, 2004). Similarly, in osteogenesis Wnt signalling has been reported to be positive (Bennett et al, 2005; Gaur et al, 2005), negative (Boland et al, 2004; Cho et al, 2006) or stage dependent (Eijken et al, 2008; Kahler et al, 2006). In chondrogenesis, similar complexities are also evident (Etheridge et al, 2004; Hartmann & Tabin, 2001; Tuli et al, 2003).

Sir2-related proteins or sirtuins are highly conserved NAD-dependent deacetylases that were shown to regulate lifespan in lower organisms (Tissenbaum & Guarente, 2001; Viswanathan & Guarente, 2011) and affect diseases of aging in mammals, such as diabetes, inflammation and neurodegenerative diseases (Donmez & Guarente, 2010). The Sir2 ortholog SIRT1 is known to deacetylate numerous mammalian transcription factors important for aging and disease (Guarente, 2011; Imai et al, 2000). Transgenic mice with roughly twofold higher levels of SIRT1 expression globally are protected against metabolic decline due to aging (Banks et al, 2008; Bordone et al, 2007; Herranz et al, 2010). Importantly, these mice are also protected against aging-induced bone loss (Herranz et al, 2010). Low to moderate SirT1 overexpression also protects mice from paraquat-induced cardiac stress and apoptosis, and delays the onset of age-dependent heart muscle dysfunctions (Alcendor et al, 2007).

Conversely, mice with reduced SIRT1 levels show defects. Sirt1^+/−^ heterozygous female mice exhibit a reduction in bone mass characterized by decreased bone formation and increased marrow adipogenesis (Cohen-Kfir et al, 2011). SirT1 heterozygotes also show increased osteoarthritis and increased levels of chondrocyte apoptosis in cartilage (Gabay et al, 2012). In SIRT1 muscle specific knock-out mice, endurance, electron transport chain activity and voluntary wheel running-induced mitochondrial biogenesis are not impaired, but responses of these same parameters to calorie restriction are severely compromised (Philp et al, 2011; Schenk et al, 2011).

As SIRT1 levels affect bone, fat, cartilage and muscle, we investigated the role of SIRT1 in the common precursor cells, MSCs, by creating MSC specific SIRT1 knock-out (MSCKO) mice. Our findings reveal important roles of SIRT1 in MSC function.

## RESULTS

### Phenotypes of MSCKO mice

**Figure 1:**
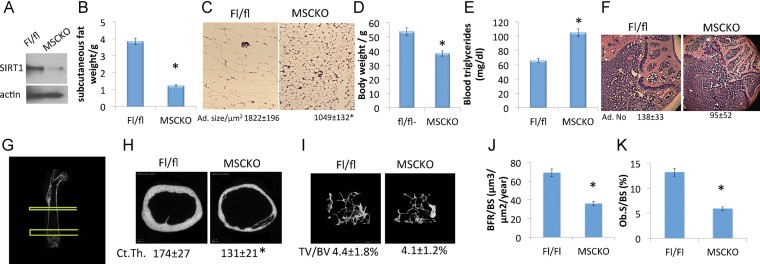
The role of SIRT1 in mesenchymal progenitor cells in aged (2.2 years old) mice *in vivo* **A.** Western blot analysis of SIRT-1 in mesenchymal progenitor cells from the bone marrow.**B.** Subcutaneous fat weight of fl/fl and MSCKO mice. Bars, SD; *p = 0.01, *t*-test, *n* = 8 per group.**C.** Haemalaun–eosin staining of subcutaneous adipose tissue, 20× magnification.**D.** Body weight of fl/fl and MSCKO mice. Bars, SD; *p = 0.006 *versus* fl/fl, *t*-test, *n* = 8 per group.**E.** Blood tryglyceride level in fl/fl and MSCKO mice. Bars, SD; *p = 0.04, *t*-test, *n* = 8 per group.**F.** Bone marrow histology of fl/fl and MSCKO mice (haemalaun–eosin staining, 5× magnification).**G–I.** Longitudinal microCT image (**G**) through femur depicting areas of midshaft and distal femur analysed in experiments. MicroCT images of the (**H**) midshaft femur (left) and (**I**) distal femur, Ct.Th, cortical thickness; TV/BV trabecular volume/bone volume; results are mean ± SD; *p = 0.01, *t*-test, *n* = 6 per group.**J, K.** Histomorphometry of femurs. (**J**) BFR/BS, bone formation rate/bone surface, Bars, SD; *p = 0.02, *t*-test, *n* = 6 per group; (**K**) ObS/BS, osteoblast surface/bone surface, Bars, SD; *p = 0.04, *t*-test, *n* = 6 per group.

**Figure 2:**
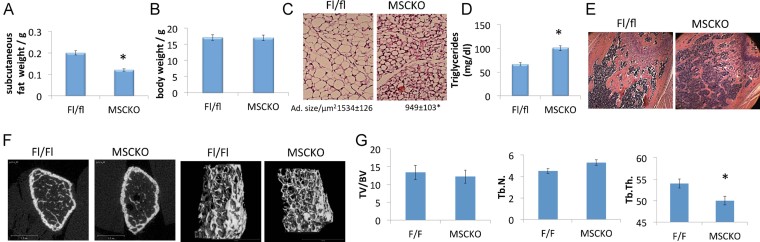
The role of SIRT1 in mesenchymal progenitor cells in young (2-month-old) mice *in vivo* **A, B.** Subcutaneous fat (**A**) and overall body weight (**B**) of fl/fl and MSCKO mice. Bars, SD; *p* = 0.05, *t*-test, *n* = 6 per group.**C.** Haemalaun–eosin staining of subcutaneous adipose tissue, 20× magnification.**D.** Blood tryglyceride level in fl/fl and MSCKO mice. Bars, SD; *p* = 0.04, *t*-test, *n* = 6 per group.**E.** Bone marrow histology of fl/fl and MSCKO mice (haemalaun–eosin staining, 5× magnification).**F.** MicroCT images of the distal femur, transversal section on the left, 3D longitudinal reconstruction on the right.**G.** MicroCT analysis of distal femur. TV/BV, trabecular volume/bone volume, Tb.N, trabecular number, Tb.Th. trabecular thickness, Bars, SD; *p = 0.05, *t*-test, *n* = 6 per group.

To test the role of SIRT1 in mesenchymal progenitor cells we generated MSC specific SIRT1 knock-out (MSCKO) mice by crossing mice with cre recombinase expression driven by the Prx-1 promoter (Logan et al, 2002) with SIRT1 floxed exon 4 mice (Cheng et al, 2003; Fig 1A). Prx-1 is a homeobox gene expressed in mesenchymal progenitor cells and is important in limb growth (Calo et al, 2010; Logan et al, 2002). All mice were in a pure C57BL/6 background. We analysed both young (2-month-old) and aged (2.2 years old) MSCKO and wild-type (fl/fl) mice. Aged MSCKO mice had a 64% reduction in the subcutaneous fat weight (Fig 1B) with smaller adipocytes (Fig 1C), and an overall reduction of 29% in body weight (Fig 1D). There was no change in visceral fat, consistent with the fact that there was no excision of SIRT1 in the visceral fat compartment (Supporting Information Fig S1A–C). However, total adipose tissue volume was decreased (Supporting Information Fig S1C). Blood tryglycerides were increased (Fig 1E) and cholesterol level was unchanged in MSCKO mice (Supporting Information Fig S2A). MSCKO mice had no signs of fatty liver disease, but had increased fasting glucose, suggestive of metabolic syndrome (Supporting Information Fig S2B and C). Cortical bone thickness and trabecular volume/bone volume were reduced 25 and 23% (Fig 1F–I), respectively, as a result of reduced numbers of osteoblasts and a reduced bone formation rate (Fig 1J and K). However, there was no change in bone marrow adipocytes (Fig 1F). Cartilage of the long bones in MSCKO mice showed no morphological change (Supporting Information Fig S3). Muscles in MSCKO mice showed no difference by the grip strength functional test (Supporting Information Fig S4A and B). Young MSCKO mice had a less pronounced reduction of subcutaneous fat weight (35%, Fig 2A), without any change in the overall body weight (Fig 2B). However, adipocytes in the subcutaneous fat were smaller (Fig 2C), and there was an increase in blood tryglycerides (Fig 2D). The effect of SIRT1 loss on bone was also less pronounced in young mice, affecting only trabecular thickness, without a significant change of overall bone volume (Fig 2E–G).

Loss of SIRT1 from MSCs also had an impact on immune cells, which are derived from HSCs, consistent with earlier suggestions of interactions between HSCs and MSCs (Ghannam et al, 2010). We observed an increase in T-lymphocytes in blood, but not B-lymphocytes, monocytes, granulocytes or erythrocytes (Supporting Information Fig S5A). There was also an increase in the spleen size and weight in MSCKO mice (Supporting Information Fig S5B). Analysis of lymphocytes in haematopoietic organs revealed an increase in CD4^+^ lymphocytes and a decrease in T regulatory (Treg) lymphocytes in MSCKO thymus, and a decrease in Tregs in MSCKO spleen. The increase in blood T cells included both CD4^+^ and CD8^+^ lymphocytes, but there was no effect on these cells in the bone marrow (Supporting Information Fig S6).

### The effect of SIRT1 on differentiation of MSCs *in vitro*

**Figure 3:**
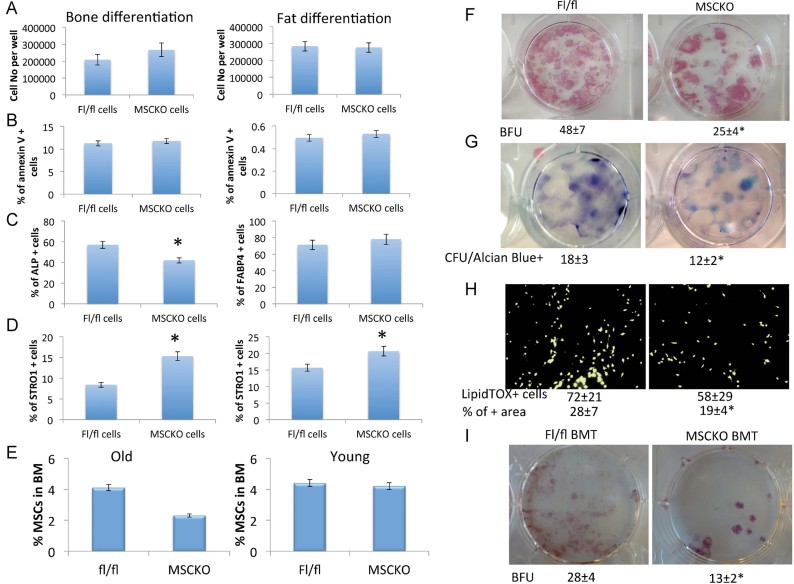
The effect of SIRT1 on differentiation of MSCs *in vitro* **A–D.** Single cell MSC proliferation and differentiation. Differentiation to osteoblasts on the left and to adipocytes on the right. (**A**) Cell number per well (*n* = 12 wells per group), (**B**) FACS analysis of apoptotic annexin V positive cells per well (*n* = 6 wells per group), (**C**) FACS analysis of ALP^+^ osteoblasts per well (*p = 0.04, *t*-test, *n* = 12 wells per group) on the left and FACS analysis of FABP4^+^ adipocytes per well (*n* = 12 wells per group) on the right, (**D**) FACS analysis of STRO-1^+^ mesenchymal progenitor cells per well (*p = 0.03 on the left and *p* = 0.05 on the right, *t*-test, *n* = 6 wells per group); bars, SD.**E.** FACS analysis of percentage of MSCs in the bone marrow of old (left, *p = 0.01, *t*-test, *n* = 6 per group) and young (right) mice.**F.** Alkaline phosphatase (ALP) staining of MSCs (isolated by the plating method) differentiated towards osteoblasts. BFU bone forming units, numbers are mean ± SD; *p = 0.001, *t*-test, *n* = 12 wells per group.**G.** Alcian blue staining of MSCs differentiated towards adipocytes. CFU/alcian blue^+^, colony forming units/Alcian blue positive, numbers are mean ± SD; *p = 0.03, *t*-test, *n* = 6 wells per group.**H.** LipidTOX staining of MSCs differentiated towards adipocytes, *p = 0.05, *t*-test, *n* = 6 wells per group.**I.** ALP staining of MSCs (isolated by the plating method) after fl/fl and MSCKO bone marrow transplantation (BMT) and differentiated towards osteoblasts. Numbers are mean ± SD; *p = 0.01, *t*-test, *n* = 12 wells per group.

Bone marrow cells of wild-type and MSCKO mice were plated to select for adherent, growing cells through reiterative cycles. This procedure has been shown to select for MSCs (Soleimani & Nadri, 2009). To demonstrate that this procedure yielded multi-potent MSCs in our hands, we performed limit dilution and placed single cells into separate wells of a 96-well plate and allowed them to proliferate. The number of total cells per well or apoptotic cells per well was not different, comparing wild-type (fl/fl) and MSCKO cells (Fig 3A and B). Cells from each well were then treated with reagents to differentiate them into osteoblasts or adipocytes. Single wild-type (fl/fl) cells were able to give rise to osteoblasts or adipocytes at a frequency between 60 and 70%, indicating that the putative MSCs isolated by this method are multi-potent (Fig 3C). Colonies from single MSCKO cells gave rise to fewer osteoblasts, and more of these cells continued to express the STRO1 MSC marker, indicating that they remained in the undifferentiated state (Fig 3D). These findings suggested that our MSCs are indeed multi-potent, but indicate a reduced ability of MSCKO cells to differentiate into osteoblasts.

To further test whether the cells we isolated were multi-potent, we inserted a GFP-expressing retrovirus into wild-type (fl/fl) and MSCKO derived cells, selected 15–20 cells microscopically for an MSC morphology, and injected them into early blastocysts to follow their potential to differentiate during embryogenesis. Both, wild-type (fl/fl) and MSCKO MSCs gave rise to mesenchymal tissues constituting many organs (Supporting Information Fig S7A–C). The distribution of cells in the organs was roughly similar between the groups, except for 3.5-fold more MSCKO MSCs detected in thymus, 2.4-fold more in cranium and brain and 0.5-fold less in the lungs. However, it is difficult to interpret this quantitation solely in light of differentiation potential, because it sums the abilities of injected cells to home to tissues, colonize there and differentiate.

In order to determine whether the number and differentiation potential of MSCs were affected by aging, we characterized MSCs from both young and aged mice. FACS sorting for MSCs (STRO-1^+^, glycophorin A−, CD 45−, CD 31−) from the bone marrow showed a large decrease in the MSC number in aged KO mice, but no significant change in MSC number in wild-type *versus* MSCKO young mice (Fig 3E). These findings suggest that deletion of SIRT1 promotes the loss of MSCs with aging. We next isolated MSCs from young mice by the reiterative plating method and differentiated them. Consistent with our data on singly cloned cells, we observed a reduced differentiation towards osteoblasts, and chondrocytes (48% and 33%, respectively; Fig 3F and G), in MSCKO cells compared to wild-type, but no significant difference in differentiation towards adipocytes or myocytes (Fig 3H). However, the adipocytes were smaller with less lipid as revealed by LipidTox staining (Fig 3H). Similar to the singly cloned cells above, there was no difference between wild-type and MSCKO cell proliferation (Supporting Information Fig S8).

To distinguish whether the phenotype of MSCKO MSCs is due to a cellular defect and not a systemic effect, we performed bone marrow transplantation from old fl/fl and MSCKO mice into irradiated recipient mice. Six months following the procedure, we analysed the differentiation potential of transplanted MSCs. Mice that received bone marrow from MSCKO animals had reduced differentiation of MSCs towards bone (Fig 3I), suggesting that observed phenotype of MSCKO mice is due to a differentiation defect of MSCKO MSCs. Recipient mice exhibited no difference in total body and subcutaneous fat weight nor bone and subcutaneous adipose tissue structure (Supporting Information Fig S9).

**Figure 4:**
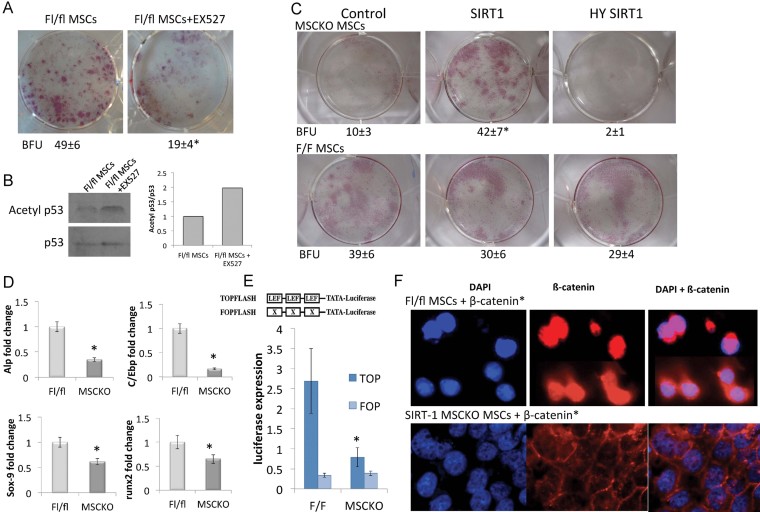
SIRT1 promotes nuclear localization of β-catenin and its activity in mesenchymal progenitor cells Alkaline phosphatase staining of fl/fl MSCs treated with SIRT1 inhibitor EX527. BFU bone forming units, mean ± SD, *p = 0.004, *t*-test, *n* = 6 wells per group.Western blot analysis of p53 acetylation as a marker of SIRT1 activity and bar graph showing acetyl53/p53 densitometry fold change.Alkaline phosphatase staining of MSCKO mesenchymal progenitor cells differentiated towards bone after transfection with control vector, SIRT1 overexpressing virus, and SIRT1 deacetylase inactive HY mutant. BFU bone forming units, mean ± SD, *p = 0.001, ANOVA Dunnett test, *n* = 6 wells per group.qPCR analysis of β-catenin targets, *n* = 4 mesenchymal progenitor samples per group, *p = 0.01–0.05, *t*-test.Luciferase reporter assays of fl/fl and MSCKO MSCs. Transcriptional activity of β-catenin in MSCs was detected using pTOP-flash (containing triple Tcf/Lef1 binding sites, the basic thymidine kinase promoter and firefly luciferase reporter gene) or pFOP-flash (containing mutated Tcf/Lef1 binding sites) plasmids and was normalized to Renilla luciferase. *p = 0.003, *t*-test, *n* = 12 wells per group, three independent experiments.Immunofluorescent staining of β-catenin and DAPI in fl/fl and MSCKO MSCs transfected with pCMV- β-cateninT41A (β-catenin*). Thirty-four plus or minus eight percent of cells showed nuclear localization in fl/fl MSCs, while 0% showed nuclear staining in MSCKO MSCs.

To test whether reduced differentiation of MSCs is due to SIRT1 activity, we treated fl/fl MSCs with the SIRT1 inhibitor EX527. *In vitro* treatment of MSCs with EX527 recapitulated the effect of MSCKO on reduced MSC differentiation (Fig 4A). The activity of EX527 was confirmed by increased acetylation of p53, a known SIRT1 target (Fig 4B).

### SIRT1 deacetylates β-catenin and promotes its nuclear localization and activity in mesenchymal progenitor cells

We next investigated the mechanism by which SIRT1 promotes osteoblast differentiation in MSCs. We first tested whether the deacetylase activity was required. Lentiviral expression of wild-type (WT) Sirt1, but not a deacetylase-inactive (H363Y) point mutant of SIRT1 (Supporting Information Fig S10), was able to restore the differentiation potential of MSCKO MSCs (Fig 4C). Microarray analysis of MSCs showed a difference in the output of the Wnt signalling pathway (Supporting Information Fig S11A) and qPCR analysis of the known target genes revealed reduction of alp, C/ebp, Sox9 and runx2 in MSCs (Fig 4D), bone, adipose tissue and cartilage of MSCKO mice (Supporting Information Fig S11B). The above findings suggested that the Wnt signalling pathway, previously implicated in MSC differentiation (see Introduction section), was defective in MSCKO cells and tissues derived from them. Analysis of additional components of the Wnt pathway revealed the increase of receptor and co-receptor, Lrp5 and Fzd1, expression, possibly by compensatory mechanisms (Supporting Information Fig S12A) and no effect on the expression of some other important bone related transcription factors like Smads (Supporting Information Fig S12B).

β-Catenin is turned over by the proteasome, when it has been phosphorylated by GSK3β Wnt functions as an extracellular ligand to trigger inhibition of GSK3β. Wnt thus leads to the stabilization and nuclear accumulation of β-catenin and the activation of target genes like runx. To further validate the defect in the Wnt pathway in MSCKO cells, we transfected a β-catenin-responsive luciferase reporter into cells, and observed a large defect in MSCKO cells compared to wild-type MSCs (Fig 4E).

**Figure 5:**
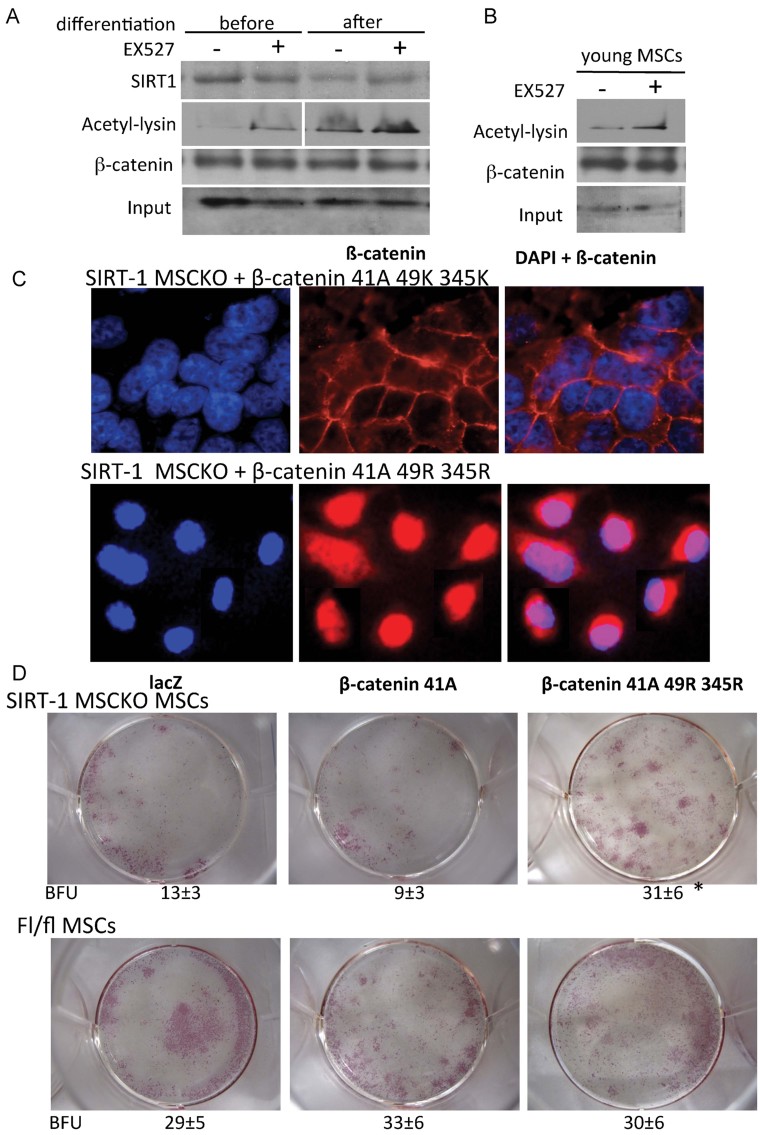
SIRT1 deacetylates β-catenin leading to nuclear localization and differentiation of MSCs Western blot analysis of old MSCs treated with SIRT1 deacetylase inhibitor EX527 before and after differentiation to osteoblasts. SIRT1 was blotted before immunoprecipitation of β-catenin and acetyl-lysine following immunoprecipitation of β-catenin.Acetylation of β-catenin in young MSCs following the treatment with SIRT1 inhibitor, EX527. MSC samples were immunoprecipitated with β-catenin antibody and membranes were probed with acetyl-lysine and β-catenin antibody.Immunofluorescent staining of β-catenin and DAPI in fl/fl and MSCKO. MSCs were transfected with the stable form of β-catenin (T41A) and a β-catenin mutant K49R K345R. Nuclear staining was 0% of cells for T41A and 29 ± 9% of cells for T41A K49R K345R.Alkaline phosphatase staining of MSCKO mesenchymal progenitor cells differentiated towards bone after transfection with the control vector, stable form of β-catenin (T41A) and β-catenin mutant where K49R, K345R. %+ area, percentage of alkaline phosphatase positive area of the well; numbers are mean ± SD, *p = 0.005 *versus* lacZ, ANOVA Dunnett test.

To locate the defect in the Wnt pathway in MSCKO cells, we transfected wild-type MSCs and MSCKO cells with a T41A mutant form of β-catenin, which resists GSK3β-mediated turnover. We thus observed β-catenin in the nucleus of WT, but not of MSCKO cells (Fig 4F). SIRT1 in MSCs deacetylated β-catenin, since reduction of its activity by EX527, SIRT1 deacetylase inhibitor, led to acetylation of β-catenin in both old and young MSCs (Fig 5A and B). In addition, when MSCs differentiate, levels of SIRT1 decrease (Fig 5A), and acetylation of β-catenin increases (Fig 5A). We further tested whether β-catenin mutations at K49 and K345 (residues previously shown to be acetylated, Lévy et al, 2004; Wolf et al, 2002) could rescue the nuclear localization defect in MSCKO cells. Indeed, transfection with β-catenin mutants K49R, K345R or the double mutant (Supporting Information Fig S13), led to nuclear localization of β-catenin in MSCKO cells (Fig 5C), and restored their potential for differentiation to osteoblasts (Fig 5D).

Finally, since β-catenin is an oncogene, we tested the tumorigenic potential of MSCs with repressed or activated β-catenin. We injected GFP marked wild-type (fl/fl), MSCKO and MSCKO expressing β-catenin K49R K345R subcutaneously into nude mice and scored for tumours. We also injected MSCs over-expressing SIRT1 (MSCTg). None of the injected mice displayed tumours. However, to our surprise MSCTg and MSCKO cells expressing K49R K345R β-catenin induced hair growth in nude mice (Supporting Information Fig S14A). All injected MSCs migrated to the hair follicle dermal papilla that has mesenchymal origin (Supporting Information Fig S14B and C). Since β-catenin signalling in dermal papilla is crucial for hair growth (Enshell-Seijffers et al, 2010), these findings further support a link between SIRT1 and β-catenin in MSCs.

## DISCUSSION

Several studies suggest an effect of SIRT1 on promoting osteogenesis and decreasing adipogenesis of MSCs (Bäckesjö et al, 2006; Li et al, 2011; Peltz et al, 2012; Puri et al, 2012; Tseng et al, 2011). Since these studies were all performed *in vitro*, we chose to investigate the role of SIRT1 in the biology of MSCs by generating MSC-specific knockout mice (MSCKO) using a floxed SIRT1 allele and a cre gene driven by the Prx-1 promoter. Our *in vivo* studies showed that loss of SIRT1 negatively impacts tissues of mesenchymal origin, especially in old mice. Defects in bone and subcutaneous fat were strikingly evident in older MSCKO mice, while milder phenotypes were found in young mice. The defects in bone in the MSCKO mice could be attributed to two effects. First, there was a significant aging-induced depletion of STRO1^+^ bone marrow cells in these mice, indicative of aging-induced MSC loss. Second, as described below, the potential of MSCs to differentiate into bone was also reduced by knocking out SIRT1. We also observed aberrations in numbers of helper and regulatory T cells in MSCKO mice, consistent with the known immunomodulatory properties of MSCs (Ghannam et al, 2010; Shi et al, 2011).

In order to study the effects of SIRT1 depletion on MSCs in more detail, we used an established protocol to isolate these cells from bone marrow (Soleimani & Nadri, 2009). Importantly, single cells isolated in this way had the potential to differentiate into bone and fat cells, indicating that they display multi-potency, one hallmark of stem cells. Injecting the putative MSCs into blastocysts also demonstrated that they were multi-potent. These MSC cells also replicated *in vitro*, indicating that they are capable of self-renewal, and no significant difference was noted between wild-type and MSCKO cells in replication. However, SIRT1 was important for the differentiation of these MSCs towards the bone and cartilage. SIRT1 was dispensable for differentiation to muscle or adipocytes, although the latter differentiated cells contained less fat and were smaller in size.

To test whether the differentiation defects in MSCKO cells was due to a reduction SIRT1-mediated deacetylation of β-catenin, we first showed that wild-type SIRT1 but not a catalytic mutant could rescue the defect in osteoblast differentiation. We further found that a β-catenin reporter was activated to a much higher level in wild-type MSCs than in MSCKO cells. Strikingly, β-catenin was immuno-localized to nuclei in the wild-type MSCs, but was extranuclear in MSCKO cells. β-Catenin is acetylated by p300 at K345 (Lévy et al, 2004) and by CREB-binding protein (CBP) acetyltransferase at K49 (Wolf et al, 2002) in epithelial cells, and is deacetylated by SIRT1 in these cells (Firestein et al, 2008). Indeed, β-catenin was hyperacetylated upon SIRT1 inhibition, and mutating K49 and K345 to arginine singly or together rescued the nuclear localization defect and bone differentiation defect in MSCKO cells. Expression of some β-catenin targets, like alp, runx2 and bmp4 is crucial for differentiation of MSCs towards bone. Expression of all these targets was reduced in MSCKO MSC-derived tissues, and this might serve as a molecular explanation for the effects on bone differentiation. However, in the case of adipose and cartilage only a subset of important factors was affected, which might explain milder phenotype in those tissues.

The above findings suggest that in MSCs, SIRT1 deacetylates β-catenin to trigger its concentration in the nucleus and activation of genes for differentiation. Intriguingly, an earlier study in epithelial cells (Firestein et al, 2008) showed that SIRT1 deacetylation of β-catenin exerts the opposite result—eviction of β-catenin from the nucleus. More generally, epithelial and mesenchymal cells show different localization of β-catenin, for example during the epithelial to mesenchymal transition (Kalluri & Weinberg, 2009). How might deacetylation of β-catenin by SIRT1 exert opposite effects in different cell types? We speculate that the nuclear import and export machinery may differ in MSCs *versus* epithelial cells, such that the deacetylated lysines in β-catenin are triggers for nuclear import in MSCs but export in epithelial cells. This would be true, for example, if both cell types had an export pathway recognizing the deacetylated lysines, but MSCs uniquely possessed an import pathway that recognizes these residues and overrides the export pathway.

In summary, we show that SIRT1 affects MSCs by deacetylating β-catenin, which increases their potential for differentiation to bone, and to a lesser extent fat. Another effect of SIRT1 is to counter the depletion of MSC number in aging mice. Our findings may represent a fraction of the biology influenced by the SIRT1/β-catenin interaction in MSCs. For example, we observed that MSCs with overexpressed SIRT1 or expressing a constitutively deacetylated β-catenin mimic had an increased potential to give rise to hair growth after injection into nude mice. It will be interesting to study the effects of SIRT1 in still other biological systems in which MSCs play a defining role.

## MATERIALS AND METHODS

### Mouse strains

Severe combined immunodeficiency mice were purchased from Charles River Laboratories. All other mice were in congenic C57Bl/6 background. MSCKO mice were generated by crossing SIRT1 allele containing a floxed exon 4 (Chen et al, 2008) with Cre-expressing mice driven by the mesenchymal progenitor cell-specific Prx1-cre promoter (Jackson Laboratory). MSCTG mice were generated by crossing a SIRT1 loxP flanked mice (Firestein et al, 2008) with mice having Prx1cre promoter. All mice were housed at 25°C and 12:12 h light/dark cycle. All experiments were performed in accordance to Massachusetts Institute of Technology guidelines and regulations and were approved by Committee on Animal Care.

### Cells and treatment

Primary mesenchymal progenitor cells were isolated from the bone marrow of tibias and femurs and then cultured with trypsinization after the cells became confluent according to previously published protocol Soleimani & Nadri, 2009). A purified population of MSCs was obtained 3 weeks after the initiation of culture. For a single cell MSC experiment one MSC for transferred into one well of 96-well plate using microscope and micropipette.

Primary MSCs were differentiated towards adipocytes using 0.5 mM isobutyl-methylxanthine (IBMX), 1 mM dexamethasone, 10 mM insulin; towards osteoblasts using 0.1 mM dexamethasone, 50 mM ascorbate-2-phosphate, 10 mM β-glycerophosphate; and towards chondrocytes using 6.25 mg/ml insulin, 10 ng/ml TGF-β1, 50 nM ascorbate-2-phosphate in α-MEM media with 10% foetal bovine serum and 1% antibiotic for 2–3 weeks (Zuk et al, 2001).

SIRT1 inhibitor EX527 (Sirtris Pharmaceuticals), was dissolved in water and was used in 10 µM concentration every other day during differentiation of MSCs.

### Plasmids and cell transfections

Stable cells were generated using lentivirus or retrovirus. The plasmids expressing mSIRT1, deacetylase inactive HY SIRT1 mutant, pBABE and PRRL-GFP vectors were purchased from Addgene. siRNA-SIRT1 plasmid has been previously described (Picard et al, 2004). The β-catenin expression vector pCMV-T41Aβ-catenin, carrying a Myc-tagged dominant stable β-catenin mutated at residue 41 (threonine to alanine), has been described previously (Wei et al, 2000). Viral production was performed by ligation of plasmid into pENTR-D-TOPO (Invitrogen) and then recombined into pLenti4-TO-V5-DEST (Invitrogen). Mutations of lysine residue K345 and/or K49 to R or A were generated in this construct with the QuickChange XL site-directed mutagenesis kit (Stratagene) and verified by sequencing. Cells were virally infected as previously described (Bell et al, 2011). Transient transfections were performed by using Fugene transfection reagent (Roche).

### Mesenchymal progenitor cell injections into blastocyst and embryo transfers

MSCs were marked with the viral transfection of GFP. Blastocysts were injected with 15–20 MCSc each using micropipette (Longenecker & Kulkarni, 2009). Blastocysts were transferred to the oviducts of pseudopregnant F1 females mated to vasectomized F1 males of proven sterility. Twelve embryos were transferred to oviduct of recipient mouse. There were four recipient mice in total, two receiving blastocysts with fl/fl MSCs and two blastocysts with MSCKO MSCs. All experiments were performed in accordance to Massachusetts Institute of Technology guidelines and regulations and were approved by Committee on Animal Care.

### Mesenchymal progenitor cell subcutaneous injections in SCID mice

MSC cells were suspended in PBS. SCID mice were anesthetized with isofluorane. We injected 10^6^ cells subcutaneously into the dorsal flank. SCID mice were sacrificed 14 days following the subcutaneous injections. All experiments were performed in accordance to Massachusetts Institute of Technology guidelines and regulations and were approved by Committee on Animal Care.

### Bone marrow transplantation

Five-week-old CD45.2 recipient mice were irradiated with a single dose of 9.5 Gy. The following day, bone marrows from fl/fl and MSCKO donor C57Bl/6 mice were injected via tail vein into recipient mice (Sharabi and Sachs, 1989). Mice were left for 6 months and bone marrow MSCs were analysed.

### MicroCT analysis of bones and adipose tissue

The microcomputerized tomography apparatus (µCT 40) and the analyzing software used in these experiments were obtained from SCANCO Medical AG (Bassersdorf, Switzerland). The distal femur was scanned in 250 slices, each 5-µm thick in the dorsoventral direction (Simic et al, 2006). Three-dimensional reconstruction of bone was performed using the triangulation algorithm. The trabecular bone volume, trabecular and cortical thickness were directly measured on three-dimensional images using the method described by Hildebrand et al (Hildebrand et al, 1999). Quantification of adipose tissue was preformed in the abdominal region from L1 to L7 using previously described method (Judex et al, 2010).

**The paper explained**

PROBLEM:

This study addresses the potential role of SIRT1 in adult MSCs, which can differentiate into osteoblasts, adipocytes, chondrocytes and myocytes.

RESULTS:

Old SIRT1 MSC-specific knockout mice showed a reduction in MSC number and defects in tissues derived from MSCs. In addition, MSCs isolated from knockout mice had a lower potential for differentiation into osteoblasts *in vitro*. This defect was associated with a defect in β-catenin function in knockout MSCs, and was rescued by mutating lysines 49 and 345 in β-catentin to arginine. We conclude that SIRT1 deacetylates β-catenin in MSCs to promote gene expression important in differentiation to osteoblasts.

IMPACT:

Our findings suggest that SIRT1 may be a therapeutic target to foster MSC differentiation to preserve integrity of MSC-derived tissues in aging animals.

### Histology, histomorphometry and immunohistochemistry

Bone, adipose, cartilage and muscle tissue histology was examined on formalin fixed, paraffin embedded sections 3 µm thick stained by haemalaun–eosin (6 sections/organ).

For histomorphometry of bones MSC fl/fl and MSCKO animals were given a subcutaneous injection of the fluorochrome calcein at 10 mg/kg (Sigma) at 4 and 14 days before death. The femurs were removed at death, prepared for histomorphometric analysis, and quantified using a computer-aided image analysis system (Bioquant II, R and M Biometrics, Nashville, TN) as previously described (Simic et al, 2006).

### Alkaline phosphatase, lipotox and alcian blue staining of MSCs

MSCs after differentiation were stained using alkaline phosphtase kit (Sigma) and LipidTOX Green Neutral Lipid Stain (Invitrogen) according to manufacturers instructions. Toluidine staining of MSCs was performed according to previously described protocol (Zuk et al, 2001). Cell size was determined using an AxioImager M1 epifluorescence microscope (Zeiss) and Image J software (version 1.37 for Mac OS X; National Institutes of Health, Bethesda, MD). In each preparation, 400 cells from various parts of the tissue slice were sized for calculation of mean cell size.

### Immunofluorescent staining

MSCs were labelled with β-catenin antibody. The slides were exposed to Alexa Fluor 488, 568 and 594 anti-rabbit and anti-mouse secondary antibodies (Invitrogen). The staining was examined with fluorescence microscope (Zeiss AxioImager.M1).

### Flow cytometric analysis and sorting

MSCs were trypsinized, resuspended in the FACS buffer and labelled with alkaline phosphatase (Abcam), FABP4 (Abcam), STRO-1 (Biolegend) and annexin V (BD) primary antibodies and Alexa Fluor 488 and Alexa Fluor 647 secondary antibodies and counted by a FACSCalibur Flow Cytometer (BD Biosciences). Haematopoietic cells were analysed using B220, Mac1, Gr1, Ter119, CD3, Lin, Kit, Sca, CD48, CD150, CD34, CD16/32, CD45 and CD31 primary antibodies and Pac Orange, Pac Blues, APC, PE-Cy7, FITC, PE and PE-Cy5 secondary antibodies. Flow cytometric analysis for marker expression was performed using FlowJo software (Ashland, OR, USA).

### RNA isolation and analysis

Total RNA from MSCs, bone marrow, adipose tissue and muscle was isolated by using Trizol (Qiagen). cDNA was synthesized from total RNA by SuperScript III reverse transcriptase (Invitrogen) with random primers. The cDNA was then subjected to real time q-PCR analysis with gene specific primers in the presence of CYBR green (Bio-Rad). Relative abundance of mRNA was normalized to rpl19.

### Western blotting

Organs and MSCs were homogenized in RIPA buffer including Complete Protease Inhibitor (Roche). Protein (100 mg) was loaded onto SDS–PAGE gels and immunoblotted with anti-SIRT1 (Upstate), β-catenin (BD Bioscience) and acetyl-lysine (Cell Signalling) antibodies.

### Luciferase assay

Transcriptional activity of β-catenin in MSCs was detected using pTOP-flash (containing triple Tcf/Lef1 binding sites, the basic thymidine kinase promoter and firefly luciferase reporter gene) or pFOP-flash (containing mutated Tcf/Lef1 binding sites) plasmids (Addgene). Luciferase activity was measured by Dual-Luciferase Reporter Assay System (Promega). The firefly luciferase activity was normalized to Renilla. The experiments were performed in triplicates and were repeated three times.

Details on microarray analysis of RNA can be found in the Supporting Information Materials and Methods.

### Statistical analysis

The analyses were performed using ANOVA Dunnett test. Differences were considered significant if *p* < 0.05.

## Supplementary material

As a service to our authors and readers, this journal provides supporting information supplied by the authors. Such materials are peer reviewed and may be re-organized for online delivery, but are not copy-edited or typeset. Technical support issues arising from supporting information (other than missing files) should be addressed to the authors.
