# SIRT1 regulates differentiation of mesenchymal stem cells by deacetylating β-catenin

**DOI:** 10.1002/emmm.201390004

**Published:** 2013-03-05

**Authors:** Petra Simic, Kayvan Zainabadi, Eric Bell, David B Sykes, Borja Saez, Sutada Lotinun, Roland Baron, David Scadden, Ernestina Schipani, Leonard Guarente

**Corrigendum to** Simic P, Zainabadi K, Bell E, Sykes DB, Saez B, Lotinun S, Baron R, Scadden D, Schipani E, Guarente L (2013) SIRT1 regulates differentiation of mesenchymal stem cells by deacetylating β-catenin. EMBO Mol Med DOI: 10.1002/emmm.201201606

The authors of the above research article regret that an error occurred during selection of images shown in Fig 4F and 5C. The images of nuclear localization of β-catenin should be replaced by the ones presented here. The previous version contained incorrectly processed images, developed purely for internal review purposes by the authors.

The conclusions of the article remain unchanged.

**Figure 1:**
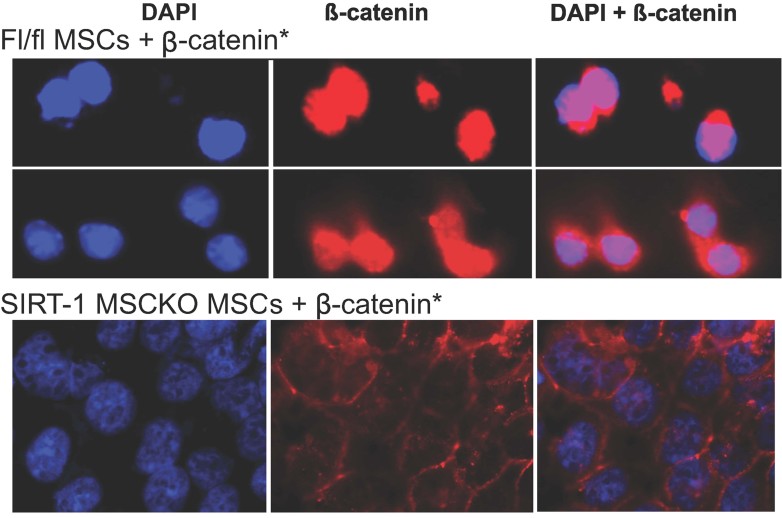
Figure 4F.

**Figure 2:**
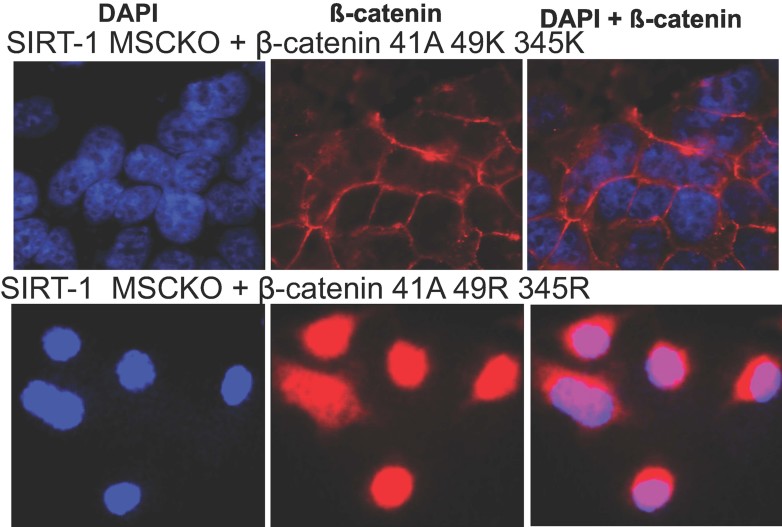
Figure 5C.

