# Supplementary material for: SIRT1 regulates differentiation of mesenchymal stem cells by deacetylating β-catenin
Source: EMBO Mol Med. 2013 Jan 30;5(3):430–40. doi: 10.1002/emmm.201201606 (PMC3598082; doi:10.1002/emmm.201201606)
Supplement: Supplementary file 1 [file emmm0005-0430-SD1.pdf]

EMBO Molecular Medicine

SIRT1 regulates differentiation of mesenchymal stem cells by deacetylating ß-catenin

Petra Simic, Kayvan Zainabadi, Eric Bell, David B. Sykes, Sutada Lotinun, Roland Baron, David Scadden, Ernestina Schipani and Leonard Guarente

Corresponding author: Leonard Guarente, MIT

Review timeline:

Submission date:	25 May 2012
Editorial Decision:	27 June 2012
Revision received:	20 August 2012
Editorial Decision:	25 September 2012
Revision received:	12 November 2012
Accepted:	14 November 2012

Transaction Report:

(Note: With the exception of the correction of typographical or spelling errors that could be a source of ambiguity, letters and reports are not edited. The original formatting of letters and referee reports may not be reflected in this compilation.)

1st Editorial Decision 27 June 2012

Thank you for the submission of your research manuscript to EMBO Molecular Medicine. We have now heard back from the three referees whom we asked to evaluate your manuscript. As you will see from the reports below, the referees find the topic of your study of potential interest. However, they raise substantial concerns on your work, which should be convincingly addressed in a major revision of the present manuscript.

I would like to particularly point out some very specific concerns regarding i) the limited mechanistic insight that would need to be strengthen (all three referees), ii) the poor KO mice phenotype description, as highlighted by both referees 2 and 3, which need to be better characterized as indicated, and iii) provide accurate experimentation, discussion and clarification when required. However, we feel that although point 2 of referee 2 would certainly be interesting, it would take too long to get the results back in time for this article. In a similar fashion, point 6 of referee 2 would of course be nice to have, but at this stage and for the purpose of this article, not mandatory.

Therefore, given the balance of these evaluations, I would like to give you the opportunity to revise your manuscript, with the understanding that the referee concerns must be fully addressed and that acceptance of the manuscript would entail a second round of review. Please note that it is EMBO Molecular Medicine policy to allow only a single round of revision and that, as acceptance or rejection of the manuscript will depend on another round of review, your responses should be as complete as possible.

Revised manuscripts should be submitted within three months of a request for revision; they will otherwise be treated as new submissions, except under exceptional circumstances in which a short extension is obtained from the editor. Also, the length of the revised manuscript may not exceed 60,000 characters (including spaces) and, including figures, the paper must ultimately fit onto optimally ten pages of the journal. You may consider including any peripheral data (but not methods

in their entirety) in the form of Supplementary information.

I look forward to seeing a revised form of your manuscript as soon as possible.

Yours sincerely,

Editor
EMBO Molecular Medicine

***** Reviewer's comments *****

Referee #1 (Other Remarks):

In this study, the investigator have partially characterized the mesenchymal phenotype of young and old MSC specific SIRT1 knock-out (MSCKO) mice. Using standard techniques, the authors report that MSCKO mice showed defects in tissues derived from MSCs including reduction in subcutaneous fat, cortical bone thickness and trabecular volume. In addition, the authors report that expression of β-catenin targets important for differentiation was reduced in MSCKO cells. The authors conclude that SIRT1 deacetylates ß-catenin to promote its accumulation in the nucleus leading to transcription of genes for MSC differentiation.

In general, this is a well written article that approaches a very interesting new subject in aging research. However, the approach to the bone phenotyping is limited and the mechanistic approach is partial.

MAJOR ISSUES:

1- The authors have made a good case assessing changes in MSC-derived tissues in this model. However, and in the particular case of bone phenotyping, the approach to some techniques is inaccurate. For instance, the quantification of alkaline phosphatase staining was not performed using the customary BFU-OB number but a somewhat imprecise %area, which is highly inaccurate.

2- Have the authors looked at changes in bone marrow fat in this model. Since in the introduction the authors gave high emphasis to the bone/fat balance, it would be interesting to see if there is switch of this model's MSC into adipogenesis.

3- A major issue of this study is the high relevance given to the accumulation of beta catenin without testing the effects of these changes on the critical elements of the osteogenic pathways. Have the authors looked at Smads, Wnt or Runx2 in these mice? This piece of information is essential to understand the implications of SIRT1 KO in these cells from a mechanistic point of view.

4- The discussion is somewhat speculative. Since this is a very interesting mouse model, the authors should discuss on the provided data. Where is the connection between SIRT1 and the osteogenic/adipogenic/chondrogenic pathways?

Overall, a more mechanistic approach is recommended and more experiments testing several elements of te MSC differentiation pathways are still required.

Referee #2 (Comments on Novelty/Model System):

The novelty of the manuscript is critically dampened by previous reports evaluating the effects of SIRT1 on MSC differentiation. Many effects described here are purely confirmatory. While those previous reports were mostly on cultured cell lines and the present article introduces a mouse model, the evaluation of the mouse model is too weak to be conclusive. The phenotyping of the mice is poor and the effects might not stem from lineage differentiation processes.

Referee #2 (Other Remarks):

Simic and colleagues report how mice lacking SIRT1 specifically in the mesenchymal stem cell (MSC) lineage display remarkable defects in diverse tissues. In addition, the authors propose that SIRT1 influences the fate of MSC differentiation through the deacetylation of b-catenin. Overall, the study is technically well driven and provides a large amount of data. However, the concept studied is not particularly novel and most of the results presented are very preliminary and lack a clear understanding of their basis. This applies to the in vivo studies, but it is even more marked in the molecular mechanisms proposed by the authors, which are far from convincing. A list of my major criticisms can be found below:

1/ The mice where SIRT1 has been deleted in a Prx-1 dependent manner present a very noticeable phenotype. However, whether this is truly due to influences in the differentiation of bone or adipocyte lineages is not that clear to this referee. Many of the phenotypes might be explained by differential metabolic properties in the differentiated tissues rather than at the differentiation stage. In general, the metabolic phenotyping of the mice is weak. No information is provided on their lean mass, energy expenditure or locomotion. Body fat measurements are performed in a single fat depot, which might lead to error. In all, the data provided is not solid enough to conclude that alterations in lineage differentiation really occur at all.

2/ It would mandatory to perform bone marrow transplantation tests crossing WT and MSCKO mice in order to solidify the authors' hypothesis.

3/ The authors should provide molecular evidence explaining why the effects of SIRT1 deletion are lineage-specific.

4/ The authors should try to knock-down SIRT1 in MSCs and evaluate whether this mimics the effects of the knock-out in their potential for differentiation.

5/ The link between SIRT1, b-catenin signaling and MSC differentiation is attractive, but far from solid. The authors should: a) demonstrate that b-catenin acetylation is dynamically regulated during specific lineage differentiation processes and how this is crucially affected by SIRT1 deletion; b) demonstrate that changes in b-catenin acetylation in MSCs during ageing might actually explain the defects observed in the MSCKO mice; c) evaluate whether b-catenin signaling is also affected specifically in tissues derived from MSCKO cells. Altogether, the physiological modulation of b-catenin acetylation should be exquisitely scrutinized in this model to fully demonstrate the authors' hypothesis

6/ Therapeutic strategies based on the authors' hypothesis should be experimentally tested. From a pharmacological perspective, do SIRT1 activating compounds influence MSC differentiation? If so, does this happen in a SIRT1 dependent manner?

Minor comments:
-The transgenic mice are randomly referenced as MSCKO and MSKO. The authors should reference them in a uniform fashion.
-The article has many data that does not add value to the article unless the molecular mechanisms are clearly nailed. This is the case, for example, of the final supplementary figures.

Referee #3 (Other Remarks):

In this interesting report, Simic and colleagues show that mice lacking Sirt1 in the mesenchimal stem cells show deffects in the tissues derived from them as they age. They propose beta-catenin as the mechanistic link, showing lower activation and lower expression of its targets, which correlates with deficient differentiation potential in vitro, and this is nicely rescued with a mutant beta-catenin, which cannot be acetylated.
Sirt1 involvement in protection from bone loss is not novel, and has been previously shown in both transgenic and deficient mouse models for sirt1,as the authors state.
However, the approach used by the authors is novel, and clearly demonstrates an important role for Sirt1 in MSCs. Overall, i think it is an important study which should be suitable for publication after the authors answer positively the following points:

© EMBO

Major points:

1.The authors propose beta-catenin as the mechanistic link, and this is supported by the rescue experiments with Sirt1 wt/mutant and beta-catenin wt/mutant. However, the authors don't show the endogenous levels of acetylated beta-catenin in the nucleous of fl/fl and Sirt1-KO MSCs.
I think it is necessary that the authors show this data to support unequivocally the mechanistic acetylation link, even more given the previous conflicting report by Firenstein et al.
The authors can perform this analysis preferentially by IP-WB of uninfected MSCs but, if this would prove difficult to detect, thay can also perform this experiment on the MSCs infected with WT beta-catenin.

Minor points:

1.The authors state that "microarray analysis of MSCs showed significant reduction in the output of Wnt singaling pathway in MSKO cells", yet they don't show this data.
Microarray data should be added to the manuscript. Is Wnt signaling the most downregulated pathway? Or do they see other (more) important changes?

2.These mice show phenotypes regarding fat storage (less) and TG output (more) both at the young and old ages. This is an interesting finding. Are these mice protected from diabetes/cardiovascular diseases onset? Or more prone to them?
Have the authors performed Glucose Tolerance Tests in young/old mice? Or fed them under HFD?
If not, it would be interesting to at least explore the accumulation of fat in the liver of the old mice, and check whether there is any change in between genotypes.
Authors might discuss the contribution of MSCs to the development of metabolic disorders.

3.In fig. S1, why there is still some Sirt1 detectable in the MSCKO?
Deacreased Sirt1 levels (together with acetylated beta-catenin levels asked for in the Major Point 1) should be shown in the main figures.

4.In figure 3E the authors conclude that "Sirt1 deletion promotes a more rapid loss of MSCs with aging". However, there is no aging associated decrease in the case of the WT MSCs, since both young and old mice show the same % of MSCs in BM. This is striking. MSCs don't decrase with aging? Is this conflicting with previous reports? Authors should discuss this issue. In any case, it would just mean that Sirt1 protects from losing MSCs so they would need to re-write the sentence deleting "more rapid".

5.In figure 4b authors state "runx levels" in the text while they show "Alp levels" in the figure. Please clarify.

6.Figure 4B and 4C are missing their p-values, please add them.

1st Revision - authors' response 20 August 2012

Referee #1:

We wish to thank this reviewer for constructive comments that helped to improve our manuscript. Here are our are point by point responses:

MAJOR ISSUES:
1- The authors have made a good case assessing changes in MSC-derived tissues in this model. However, and in the particular case of bone phenotyping, the approach to some techniques is inaccurate. For instance, the quantification of alkaline phosphatase staining was not performed using the customary BFU-OB number but a somewhat imprecise %area, which is highly inaccurate.

We thank the reviewer for pointing this out, and we now use BFU number to quantify the alkaline

phosphatase staining.

2- Have the authors looked at changes in bone marrow fat in this model. Since in the introduction the authors gave high emphasis to the bone/fat balance, it would be interesting to see if there is switch of this model's MSC into adipogenesis.

There is no difference in bone marrow adipogenesis. Number of adipocytes in the bone marrow is now shown in Fig 1E.

3- A major issue of this study is the high relevance given to the accumulation of beta catenin without testing the effects of these changes on the critical elements of the osteogenic pathways. Have the authors looked at Smads, Wnt or Runx2 in these mice? This piece of information is essential to understand the implications of SIRT1 KO in these cells from a mechanistic point of view.

We have now analyzed the expression of additional components of Wnt pathway, like ligands, receptors and co-receptors. There is a compensatory increase in the Lrp5 and Frz1 expression in MSCKO MSCs (Supplementary Figure 10). Runx2, b-catenin downstream target, expression is decreased (Fig 4B), additionally confirming the effect on osteogenic pathway. Importantly there was no significant effect on Smads (Supplementary Figure 10).

4- The discussion is somewhat speculative. Since this is a very interesting mouse model, the authors should discuss on the provided data. Where is the connection between SIRT1 and the osteogenic/adipogenic/chondrogenic pathways?
Overall, a more mechanistic approach is recommended and more experiments testing several elements of the MSC differentiation pathways are still required.

We have incorporated into the discussion additional data on b-catenin targets and discuss other transcription factors in MSCs and MSC-derived tissues that may explain the skewing of effects toward bone.

Referee #2

We wish to thank the reviewer for extremely helpful criticism. We were able to incorporate all the suggestions.

Comments on Novelty/Model System:

The novelty of the manuscript is critically dampened by previous reports evaluating the effects of SIRT1 on MSC differentiation. Many effects described here are purely confirmatory. While those previous reports were mostly on cultured cell lines and the present article introduces a mouse model, the evaluation of the mouse model is too weak to be conclusive. The phenotyping of the mice is poor and the effects might not stem from lineage differentiation processes.

Previous reports were only on in vitro isolated bone marrow cells with somewhat conflicting data on differentiation and proliferation of MSCs and are discussed in details in the Discussion section. This is the first in vivo analysis of the effect of SIRT1 on MSCs. Now, we characterized phenotype in more details, including bone marrow fat and overall body fat and provide more data on lineage specific differentiation effects.

Referee #2 (Other Remarks):

1/ The mice where SIRT1 has been deleted in a Prx-1 dependent manner present a very noticeable phenotype. However, whether this is truly due to influences in the differentiation of bone or adipocyte lineages is not that clear to this referee. Many of the phenotypes might be explained by differential metabolic properties in the differentiated tissues rather than at the differentiation stage. In general, the metabolic phenotyping of the mice is weak. No information is provided on their lean mass, energy expenditure or locomotion. Body fat measurements are performed in a single fat depot,

which might lead to error. In all, the data provided is not solid enough to conclude that alterations in lineage differentiation really occur at all.

Micro-CT analysis of adipose tissue of Fl/fl and MSCKO mice was performed and has revealed a decrease in subcutaneous fat and no change in visceral fat, leading to a decrease in total adipose tissue volume. This is now shown in Supplementary Figure 1C.

2/ It would be mandatory to perform bone marrow transplantation tests crossing WT and MSCKO mice in order to solidify the authors' hypothesis.

Fortunately, we did have this data and now show the results of fl/fl and MSCKO bone marrow transplantation into irradiated recipient mice. Six months following the transplantation isolated MSCs show significant reduction in bone nodule formation (Figure 3I).

3/ The authors should provide molecular evidence explaining why the effects of SIRT1 deletion are lineage-specific.

We have analyzed additional b-catenin targets in different MSC derived tissues which might be responsible for lineage-related differentiation defects (Fig S11B). We found broader effects on bone targets compared to fat and cartilage, which may help explain the specificity.

4/ The authors should try to knock-down SIRT1 in MSCs and evaluate whether this mimics the effects of the knock-out in their potential for differentiation.

We have treated MSCs with the very specific and potent SIRT1 inhibitor EX527 and were able to mimic the effect of MSCKO in vivo (Figure 4A and B).

5/ The link between SIRT1, b-catenin signaling and MSC differentiation is attractive, but far from solid. The authors should: a) demonstrate that b-catenin acetylation is dynamically regulated during specific lineage differentiation processes and how this is crucially affected by SIRT1 deletion; b) demonstrate that changes in b-catenin acetylation in MSCs during ageing might actually explain the defects observed in the MSCKO mice; c) evaluate whether b-catenin signaling is also affected specifically in tissues derived from MSCKO cells. Altogether, the physiological modulation of b-catenin acetylation should be exquisitely scrutinized in this model to fully demonstrate the authors' hypothesis

Now we show that reduction of SIRT-1 activity in MSCs leads to b-catenin acetylation and that level of acetylation increases upon differentiation (Fig 5A). The increasing level of b-catenin acetylation correlates with the decrease of SIRT-1 levels upon differentiation (Fig 5A). There is a difference in acetylation of b-catenin in MSCKO animals as well (Fig 5B). Finally, b-catenin signaling is affected in MSC derived tissues as well, as shown by qPCR analysis of b-catenin targets in the bone, fat and cartilage (Fig S11B).

6/ Therapeutic strategies based on the authors' hypothesis should be experimentally tested. From a pharmacological perspective, do SIRT1 activating compounds influence MSC differentiation? If so, does this happen in a SIRT1 dependent manner?

Since we only tested deletion and not over-expression in vivo, it seems most appropriate to examine effects of SIRT1 inhibitors. We thus tested the SIRT1 inhibitor EX527, as discussed above (Fig 4A, B). We agree that it will be important to examine the effects of SIRT1 activation in the future.

Minor comments:

-The transgenic mice are randomly referenced as MSCKO and MSKO. The authors should reference them in a uniform fashion.

Now all mice are referenced as MSCKO.

The article has many data that does not add value to the article unless the molecular mechanisms are clearly nailed. This is the case, for example, of the final supplementary figures.

We believe the data in this version is all of value to the manuscript.

Referee #3 (Other Remarks):

In this interesting report, Simic and colleagues show that mice lacking Sirt1 in the mesenchimal stem cells show deffects in the tissues derived from them as they age. They propose beta-catenin as the mechanistic link, showing lower activation and lower expression of its targets, which correlates with deficient differentiation potential in vitro, and this is nicely rescued with a mutant beta-catenin, which cannot be acetylated.
Sirt1 involvement in protection from bone loss is not novel, and has been previously shown in both transgenic and deficient mouse models for sirt1,as the authors state.
However, the approach used by the authors is novel, and clearly demonstrates an important role for Sirt1 in MSCs. Overall, i think it is an important study which should be suitable for publication after the authors answer positively the following points:

Major points:
1.The authors propose beta-catenin as the mechanistic link, and this is supported by the rescue experiments with Sirt1 wt/mutant and beta-catenin wt/mutant. However, the authors don't show the endogenous levels of acetylated beta-catenin in the nucleous of fl/fl and Sirt1-KO MSCs.
I think it is necessary that the authors show this data to support unequivocally the mechanistic acetylation link, even more given the previous conflicting report by Firenstein et al.
The authors can perform this analysis preferentially by IP-WB of uninfected MSCs but, if this would prove difficult to detect, they can also perform this experiment on the MSCs infected with WT beta-catenin.

Now we show endogenous levels of b-catenin and its acetylation in MSCs as a function of SIRT1 activity in Figures 4B, 5A and 5B.

Minor points:

1.The authors state that "microarray analysis of MSCs showed significant reduction in the output of Wnt singaling pathway in MSKO cells", yet they don't show this data.
Microarray data should be added to the manuscript. Is Wnt signaling the most downregulated pathway? Or do they see other (more) important changes?

Gene set enrichment analysis of microarray data is now included in Fig S11A. There are other significantly changed pathways, but we focused on Wnt signaling because of the relationship to all MSC-derived tissues.

2.These mice show phenotypes regarding fat storage (less) and TG output (more) both at the young and old ages. This is an interesting finding. Are these mice protected from diabetes/cardiovascular diseases onset? Or more prone to them?
Have the authors performed Glucose Tolerance Tests in young/old mice? Or fed them under HFD? If not, it would be interesting to at least explore the accumulation of fat in the liver of the old mice, and check whether there is any change in between genotypes.
Authors might discuss the contribution of MSCs to the development of metabolic disorders.

We now show that MSCKO mice show increased fasting glucose (Fig S2B), suggestive of metabolic syndrome in the setting of decreased subcutaneous fat. Although there is an increase in blood tryglicerides, we did not observe the fatty liver disease in MSCKO mice (Fig S2C).

3.In fig. S1, why there is still some Sirt1 detectable in the MSCKO?
Deacreased Sirt1 levels (together with acetylated beta-catenin levels asked for in the Major Point 1) should be shown in the main figures.

There is some SIRT1 detectable in residual cells apart from MSCs. Western blot showing SIRT1 is now shown in the Figure 1A.

4.In figure 3E the authors conclude that "Sirt1 deletion promotes a more rapid loss of MSCs with

aging". However, there is no aging associated decrease in the case of the WT MSCs, since both young and old mice show the same % of MSCs in BM. This is striking. MSCs don't decrease with aging? Is this conflicting with previous reports? Authors should discuss this issue. In any case, it would just mean that Sirt1 protects from losing MSCs so they would need to re-write the sentence deleting "more rapid".
Discuss previous reports on number of MSCs with aging.

The conclusion of the Figure 3E is now re-written without saying "more rapid" loss of MSCs with aging. We have not observed a decrease in percentage of MSCs during aging. However, the cellularity of the bone marrow was significantly reduced in aged mice was reduced.

5.In figure 4b authors state "runx levels" in the text while they show "Alp levels" in the figure. Please clarify.

Now, we included both runx and alp levels in the Figure 4B.

6.Figure 4B and 4C are missing their p-values, please add them.

We added the p values in the Figures (now 4D and 4E).

2nd Editorial Decision 25 September 2012

Thank you for the submission of your revised manuscript "SIRT1 regulates differentiation of mesenchymal stem cells by deacetylating β-catenin" to EMBO Molecular Medicine. We have now received the reports from the reviewer who was asked to re-review your manuscript.

You will be glad to see that the reviewers are now globally supportive and we can proceed with official acceptance of your manuscript pending the minor changes detailed below:

- Please include the p53 Western blot as requested by Reviewer #3.

- For Research Articles and Reports submitted to EMBO Molecular Medicine reporting experiments on live vertebrates and/or higher invertebrates, the corresponding author must confirm that all experiments were performed in accordance with relevant guidelines and regulations. The manuscript must include a statement in the Materials and Methods identifying the institutional and/or licensing committee approving the experiments, including any relevant details.

- The description of all reported data that includes statistical testing must state the name of the statistical test used to generate error bars and P values, the number (n) of independent experiments underlying each data point (not replicate measures of one sample), and the actual P value for each test (not merely 'significant' or 'P < 0.05').

I look forward to seeing a revised version of your manuscript as soon as possible.

Yours sincerely,

Editor
EMBO Molecular Medicine

***** Reviewer's comments *****

Referee #1 (Comments on Novelty/Model System):

The revised version has integrated all the Reviewers' suggestion and therefore has been significantly improved.

Referee #1 (Remarks):

The authors have satisfactorily responded to all the Reviewer's comments. The new version of the manuscript shows additional and very interesting data.

Referee #3 (Comments on Novelty/Model System):

Authors have answered most, if not all, the reviewers concerns, therefore i think the article is suitable for publication.

I would only ask them to show p53 total levels in Fig4B, since that is the real control to test decreased p53 acetylation and, moreover, treatment with EX527 may affect the total levels of p53. Should the authors provide this data, I would have no other issue.

Referee #3 (Remarks):

Authors have addressed all my concerns.
In the new Figure 4B, authors present levels of acetyl-p53 but they don't present its total levels, which is the proper control instead of tubulin since p53 total levels are tightly regulated and might be affected by treatment with EX-527.
I would suggest that the authors show this WB.

2nd Revision - authors' response 12 November 2012

Please find enclosed revised manuscript "SIRT1 regulates differentiation of mesenchymal stem cells by deacetylating b-catenin". We have incorporated the requested changes:

- We included p53 Western blot and acetylp53/p53 densitometry bar graph in Figure 4B.
- The manuscript now includes a statement in the Materials and Methods identifying the institutional and licensing committee approving the experiments.
- All reported data that includes statistical testing now states the name of the statistical test used and the actual P value for each test

Thank you very much for your help to improve this manuscript.
