# Supplementary material for: SIRT1 regulates differentiation of mesenchymal stem cells by deacetylating β-catenin
Source: EMBO Mol Med. 2013 Jan 30;5(3):430–40. doi: 10.1002/emmm.201201606 (PMC3598082; doi:10.1002/emmm.201201606)
Supplement: Supplementary file 2 [file emmm0005-0430-SD2.pdf]

Supplementary Information

Table of Contents:

	Page
Supplementary Figure Legends	1
Supplementary Methods	3
Supplementary Figures	4

Supplementary Figure Legends

Supplementary Figure 1. SIRT-1 in subcutaneous and visceral fat. (A) RT-PCR analysis of excision of SIRT-1 in subcutaneous and visceral fat of Fl/fl and MSCKO mice. (B) Visceral fat weight of Fl/fl and MSCKO mice. Bars, SD. (C) MicroCT analysis of subcutaneous adipose tissue (SAT), visceral adipose tissue (VAT) and total adipose tissue (TAT) of abdominal region (L1-L7) of Fl/fl and MSCKO mice

Supplementary Figure 2. Additional metabolic phenotype of MSCKO *in vivo*. (A) Blood cholesterol and (B) fasting blood glucose in Fl/fl and MSCKO mice; bars, SD; * p=0.05, t-test, n=8 per group. (C) Fl/fl and MSCKO liver histology, hemalaun-eosin staining, 10x magnification.

Supplementary Figure 3. The effect of SIRT-1 on cartilage in vivo. Hemalaun-eosin staining of distal femur showing articular cartilage (10x magnification).

Supplementary Figure 4. The effect of SIRT-1 on muscle in vivo. (A) Hemalaun-eosin staining of quadriceps muscle (10x magnification). (B) Grip strength test in Fl/fl and MSCKO mice. Bars, SD.

Supplementary Figure 5. The effect of SIRT-1 MSCKO on hematopoietic cells in blood and spleen. (A) Number of B cells, monocyte/granulocyte, T cells and erythroid cells in Fl/fl and MSCKO mice; bars, SD; * p=0.05, t-test, n=6 per group. (B) Images of spleens from Fl/fl and MSCKO mice (upper panel) and spleen weight / body weight in Fl/fl and MSCKO mice (lower panel); bars, SD; * p=0.05, t-test, n=6 per group.

Supplementary Figure 6. The effect of SIRT-1 MSCKO on lymphocytes in different organs. Number of CD4 and CD8 lymphocytes, T regulatory lymphocytes (Tregs) and MSCs in thymus (* p=0.03-0.05), spleen (* p=0.003), bone marrow and blood (* p=0.04-0.05) of Fl/fl and MSCKO mice; bars, SD; t-test, n=6 per group.

Supplementary Figure 7. MSCs give rise to multiple organs following the injection into the blastocyst. (A) Distribution of GFP+ F;/fl and MSCKO MSCs at E17 following the injection into the blastocyst. (B) qPCR detection of GFP in different organs presented as the fold change as compared to Fl/fl MSCs. (C) negative control for experiment in (A) where blastocysts were injected with MSCs without the GFP.

Supplementary Figure 8. Proliferation of Fl/fl and MSCKO mesenchymal progenitor cells throughout 5 weeks. Bars SD; 24 wells per group.

Supplementary Figure 9. Phenotype of recipient mice after Fl/fl and MSCKO bone marrow transplantation (BMT). (A) Body weight and (B) subcutaneous adipose tissue (SAT) weight after Fl/fl and MSCKO BMT. bars, SD; * $p<0.05$ vs Fl/fl, t-test. (C) Histology of bone marrow (BM) and SAT of recipient mice following Fl/fl and MSCKO BMT, hemalaun-eosin staining, 10x magnification.

Supplementary Figure 10. SIRT-1 protein levels after transfection with SIRT-1 and SIRT-1 inactive deacetylase HY mutant. Western blot analysis of V5, tagged to SIRT-1 after the transfection of Fl/fl and MSCKO MSCs. Western blots were probed with V5 and tubulin antibody.

Supplementary Figure 11. Microarray and qPCR analysis of signaling pathways in MSCs and tissues derived from them. (A) Microarray gene set enrichment analysis (GSEA) of different pathways downregulated in MSCKO MSCs as compared to Fl/fl MSCs. (B) qPCR analysis of β-catenin target genes in bone, subcutaneous adipose tissue and MSCs differentiated to cartilage. bars, SD; * p=0.01-0.04, t-test, n=3 per group.

Supplementary Figure 12. Expression of genes involved in Wnt pathway. (A) qPCR analysis of ligands, receptors and co-receptors in the Wnt pathway. (B) qPCR analysis of Smads. bars, SD.

Supplementary Figure 13. β-catenin protein levels after transfection of Fl/fl and MSCKO MSCs with different β-catenin mutants. Western blots were probed with V5 (tagged to β-catenin) and tubulin antibodies.

Supplementary Figure 14. The effect of SIRT-1 level in MSCs injected subcutaneously into SCID mice on hair growth. (A) Representative picture of hair growth in SCID mice 10 days following the subcutaneous injections of Fl/fl, MSCKO, MSCKO transfected with β-catenin mutant and MSCTg MSCs. (B) Immunofluorescent staining MSCs marked with GFP in hair follicle showing co-localization with β4-integrin dermal papilla. (C) Immunohistochemistry of the femoral bone marrow using GFP antibody to GFP marked MSCs injected subcutaneously into SCID mice.

Supplementary Methods

Microarray analysis of RNA was performed using Affymetrix platform. We compared the gene expression levels from Fl/fl and MSCKO MSCs and picked up the genes which had significant different expression for Gene set enrichment analysis (GSEA) by using Molecular Signatures Database(V3.0). Gene set enrichment analysis was carried out by computing overlaps with gene sets, obtained from the Broad Institute (1). Genes in Gene Set (K), Genes in Overlap (k), k/K and P value were used to rank the pathways enriched in each phenotype. We used 39359 genes as the gene set in this study.

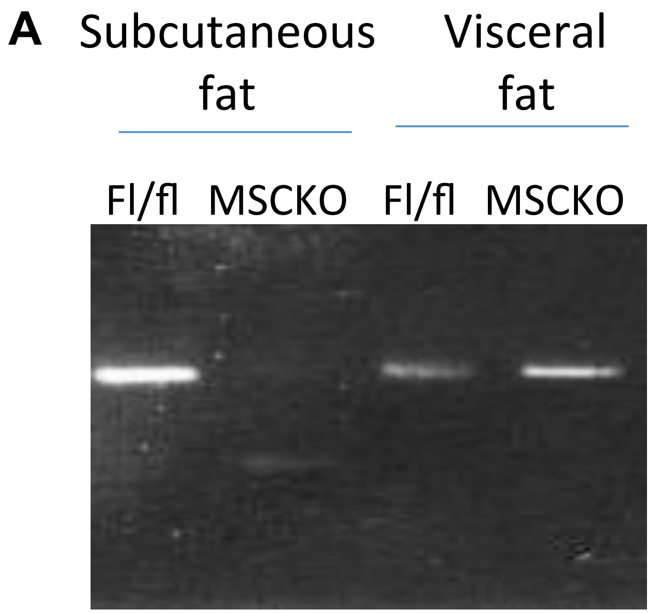

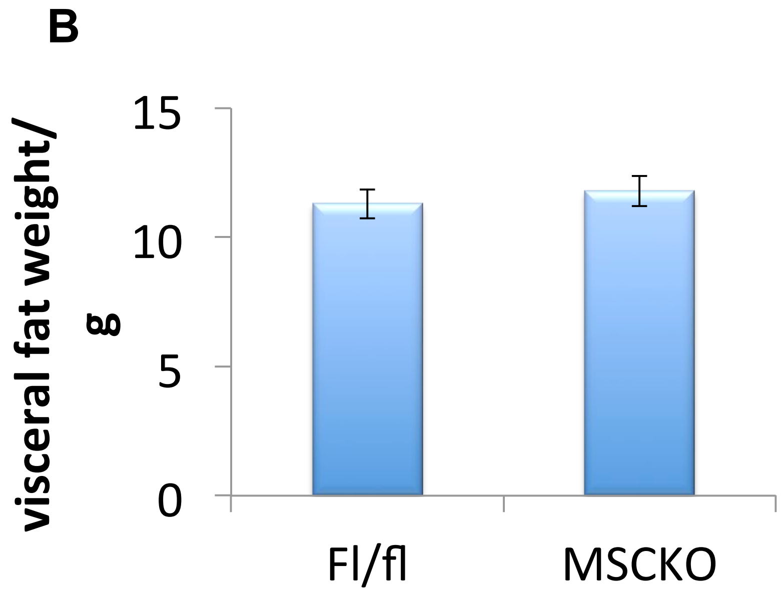

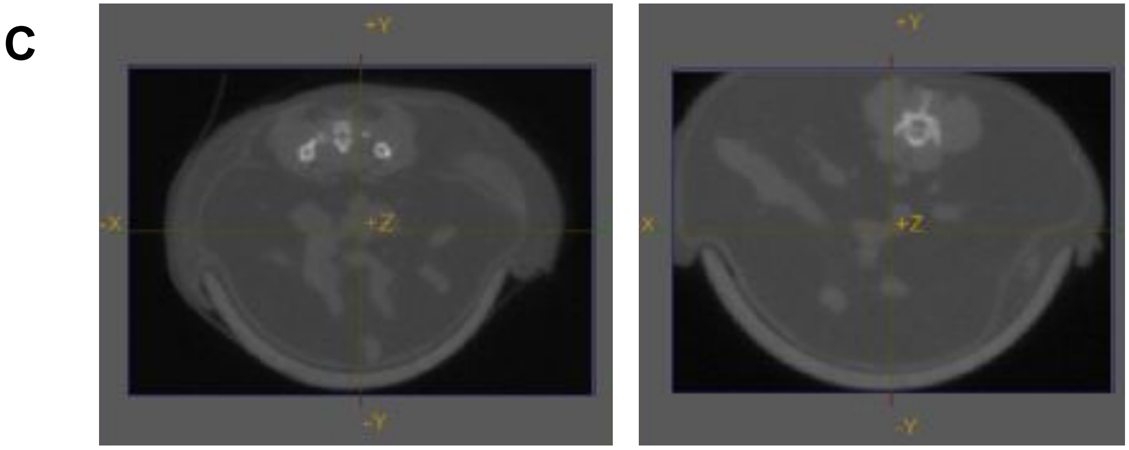

SAT/mm^3	108±28	29±5*
VAT/mm^3	751±45	698±40
TAT/mm^3	1281±120	1007±101*

Supplementary Fig 1.

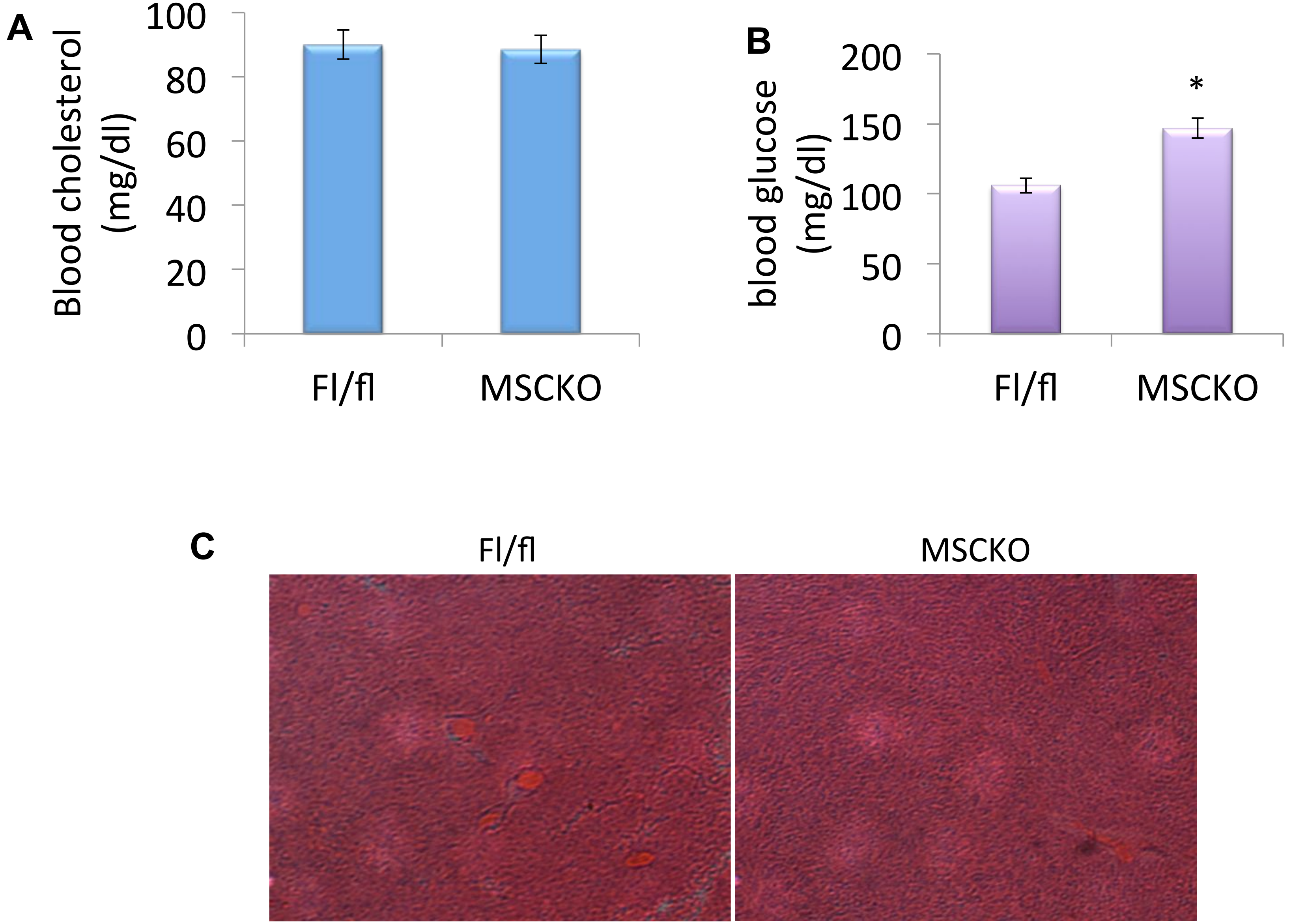

Supplementary Fig 2.

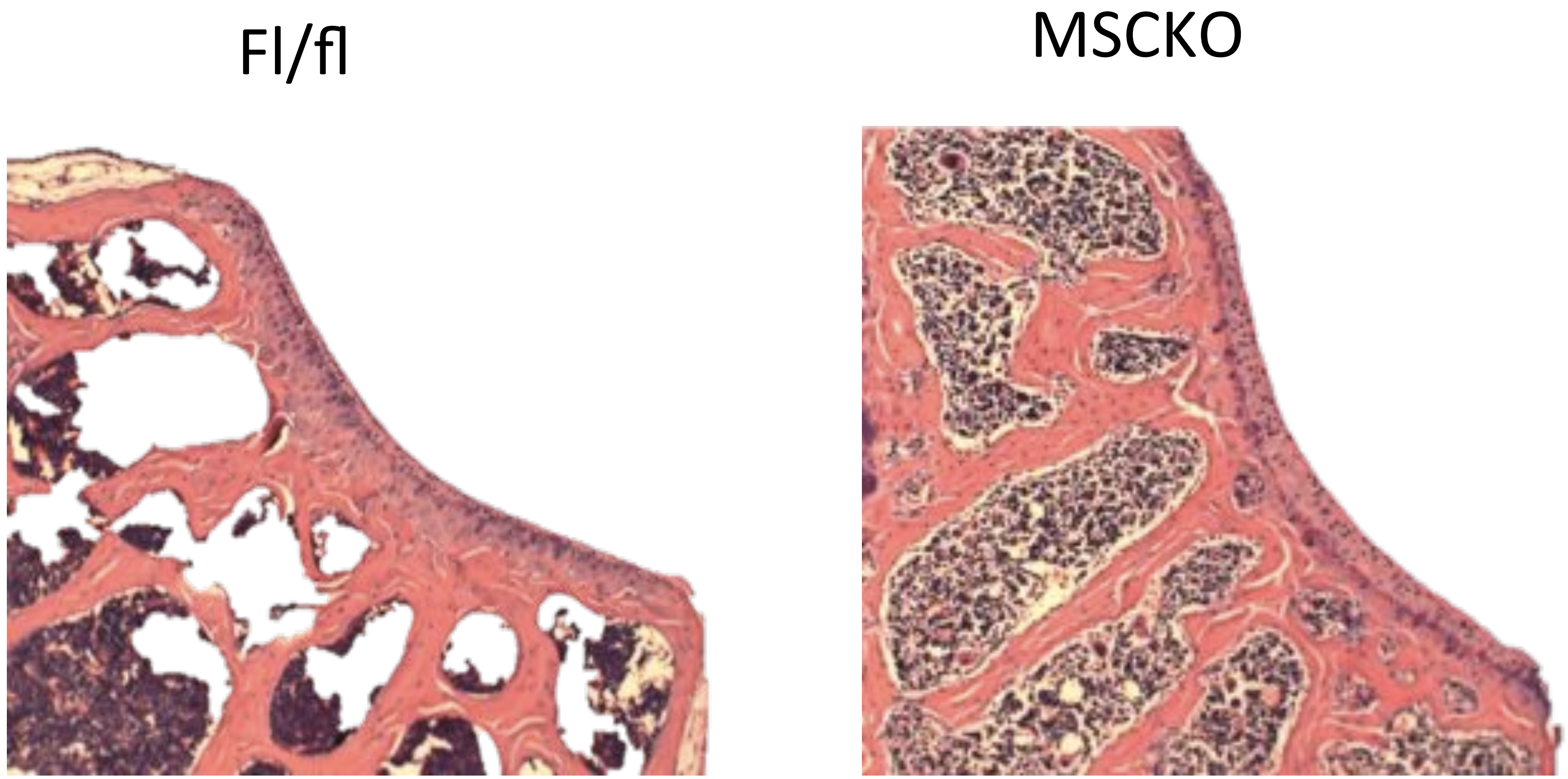

Supplementary Fig 3.

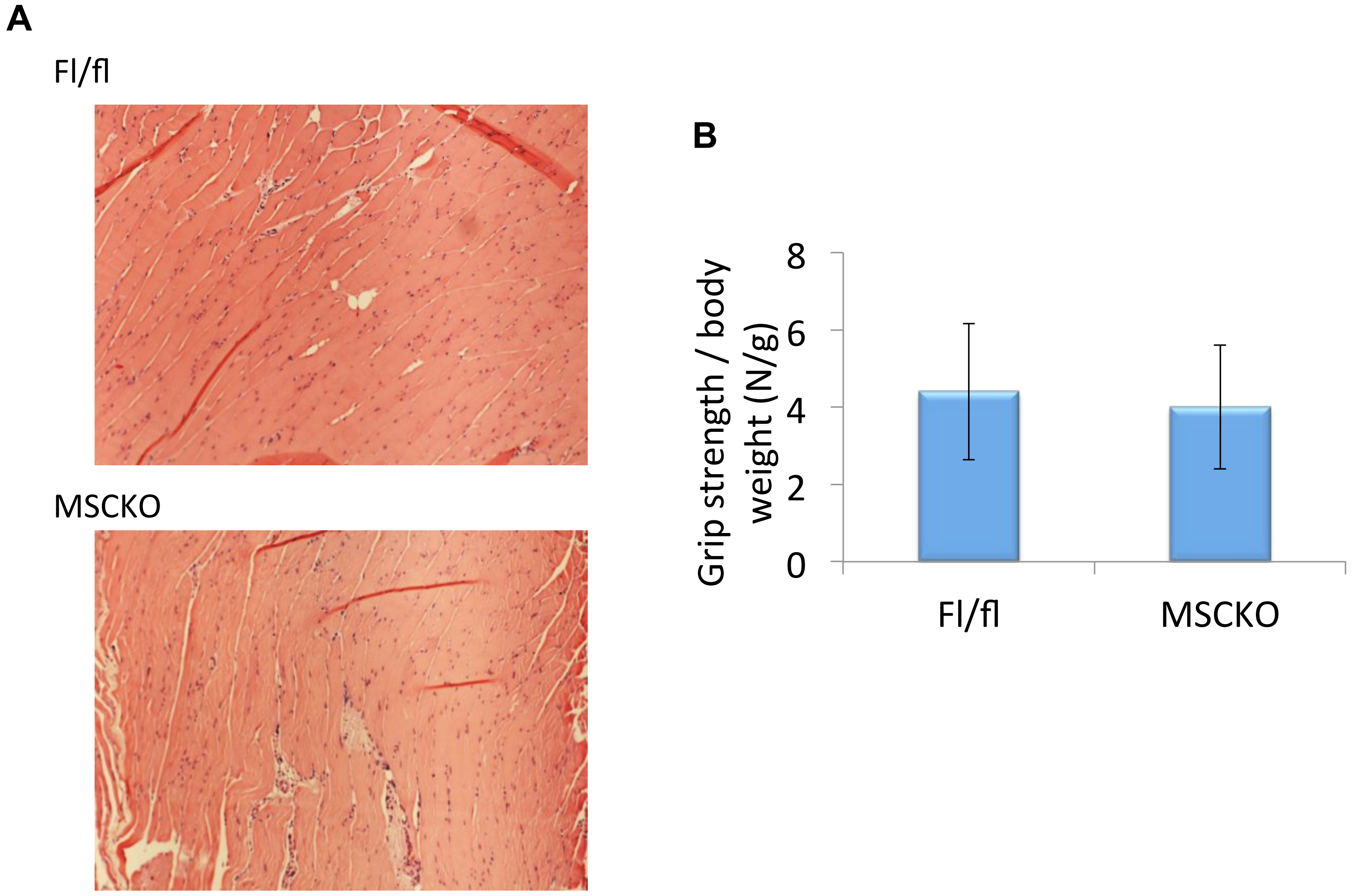

Supplementary Fig 4.

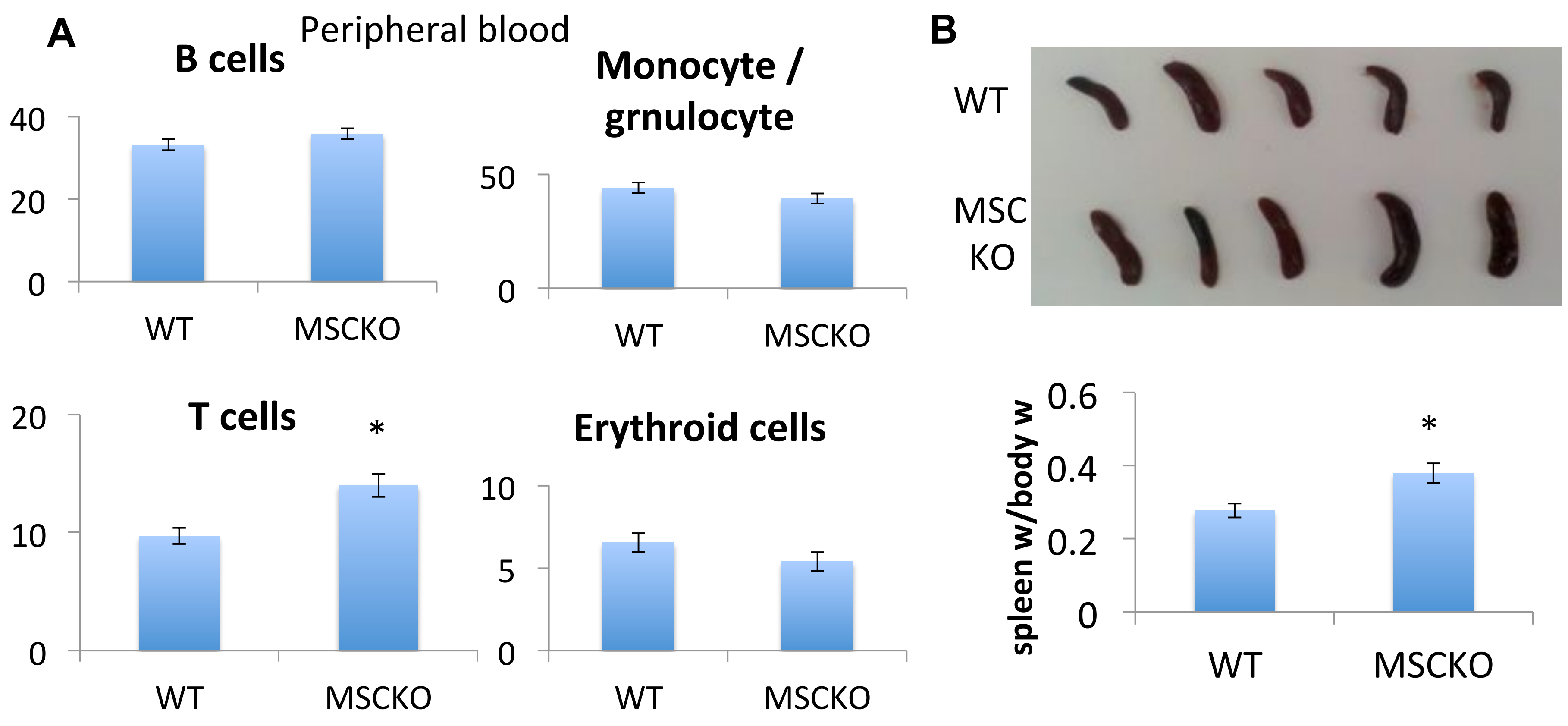

Supplementary Fig 5.

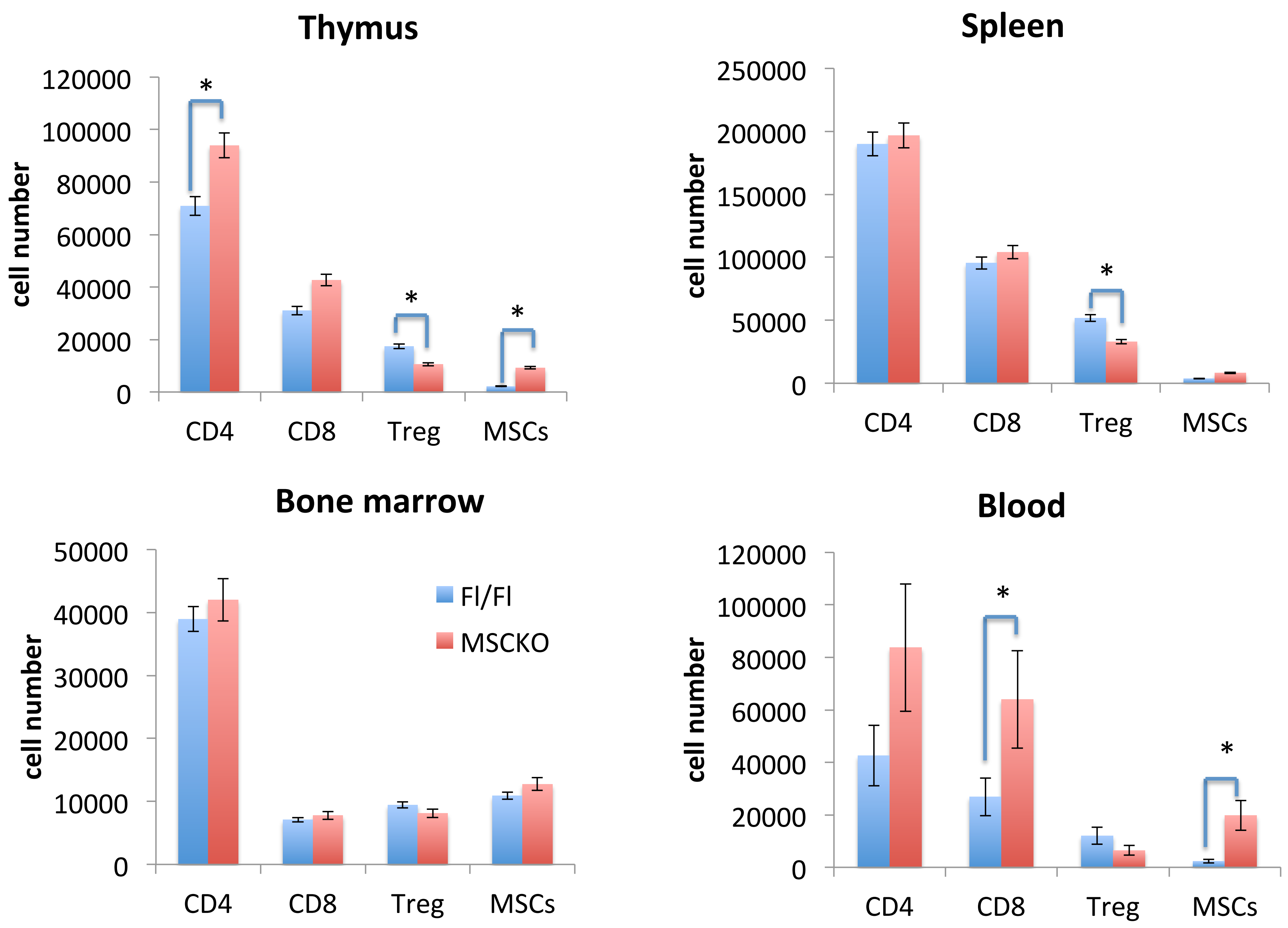

Supplementary Fig 6.

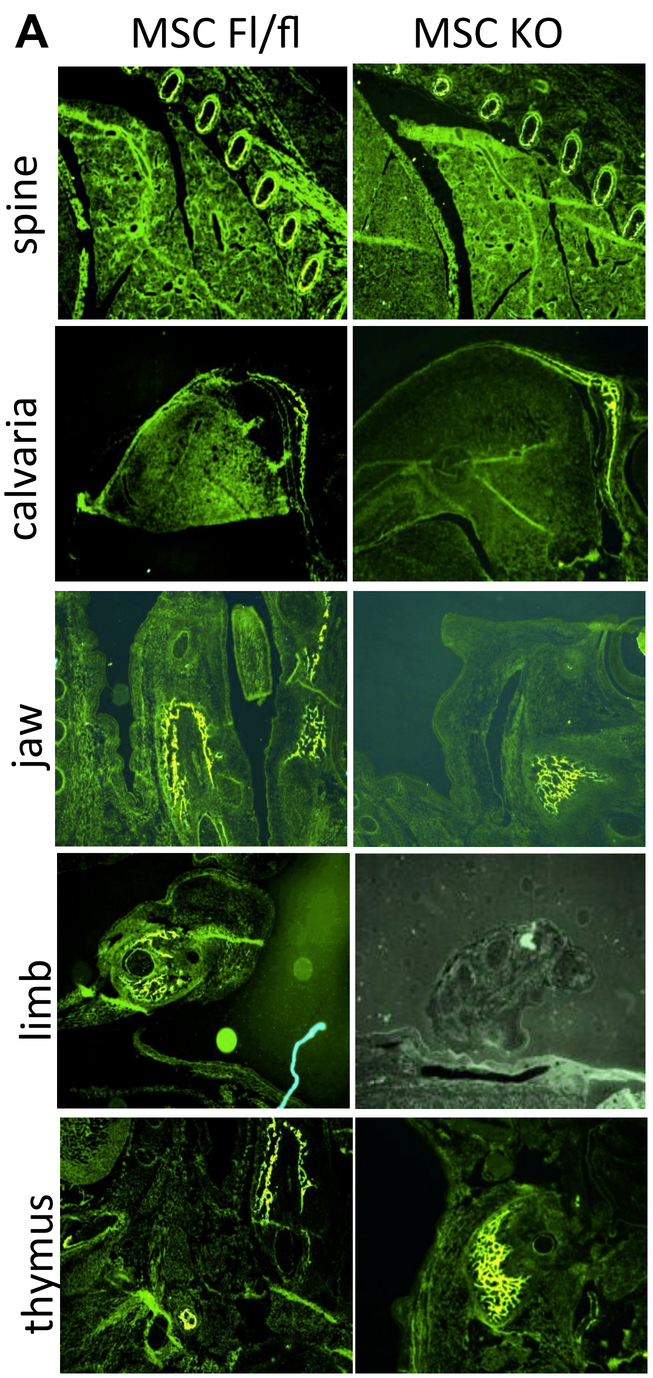

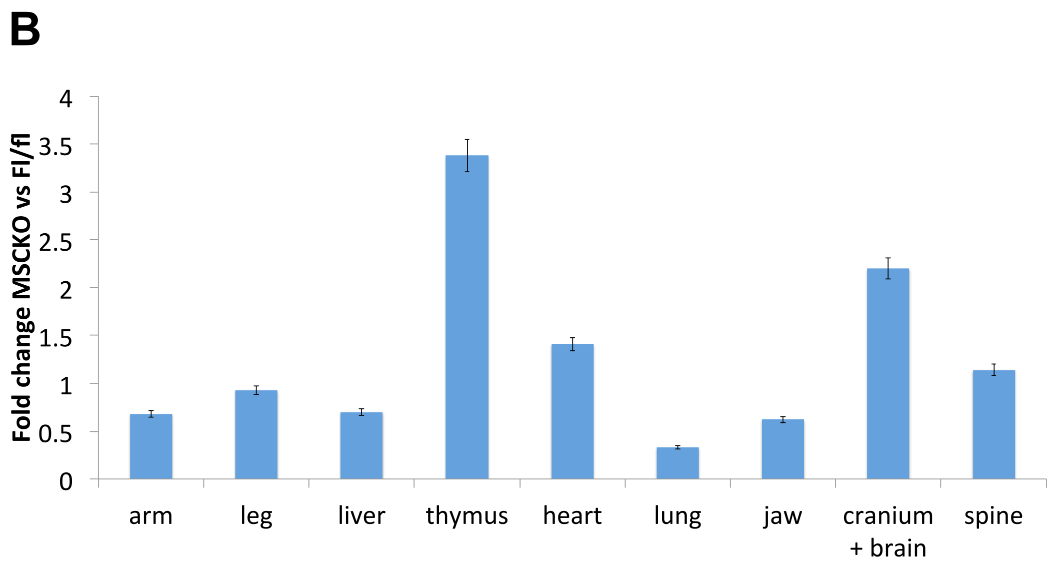

Supplementary Fig. 7.

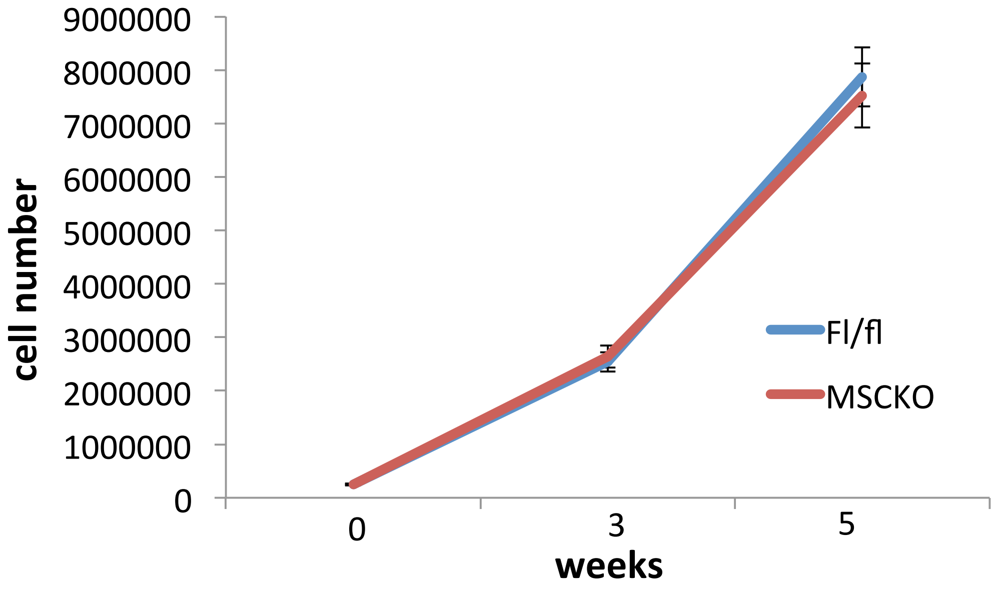

Supplementary Fig 8.

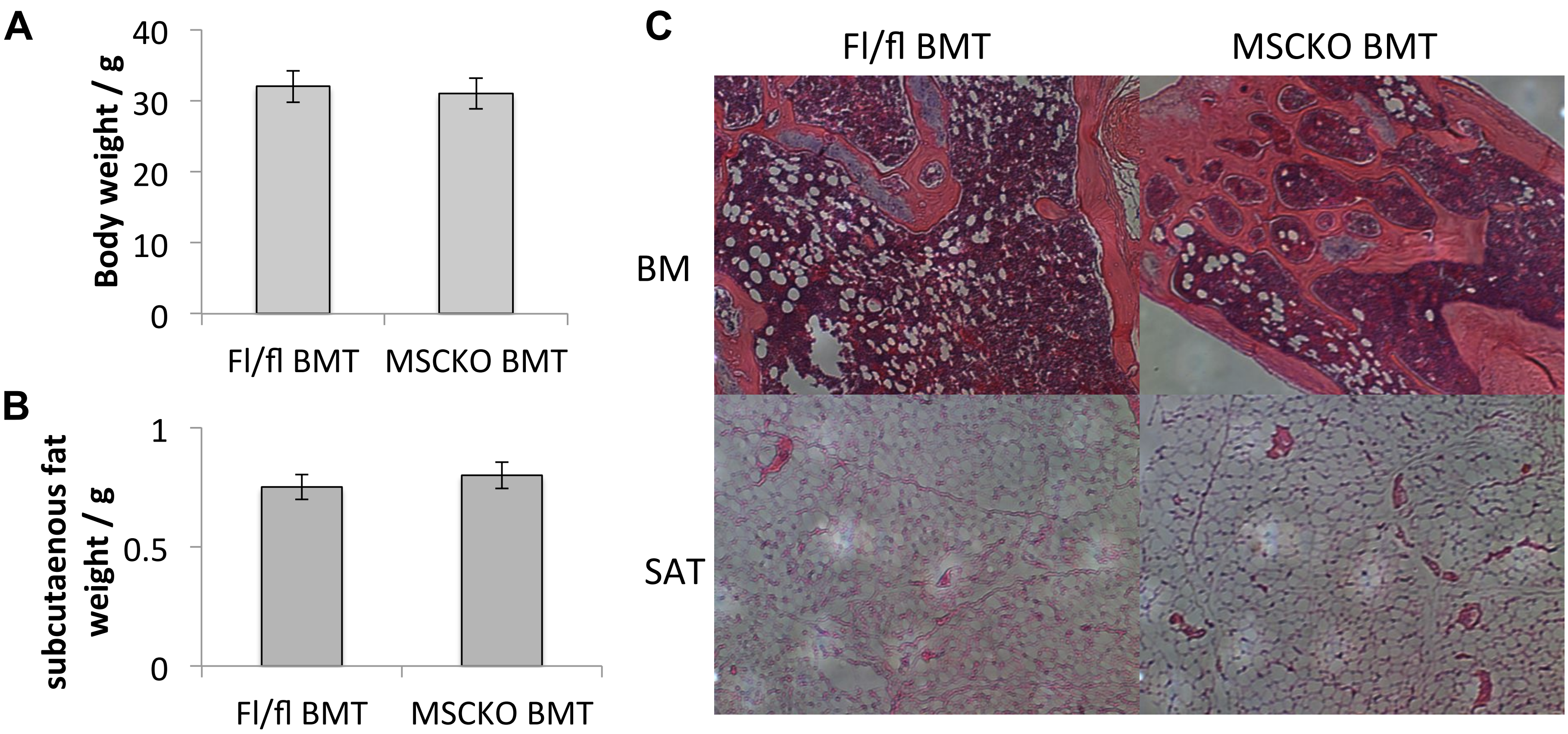

Supplementary Fig. 9.

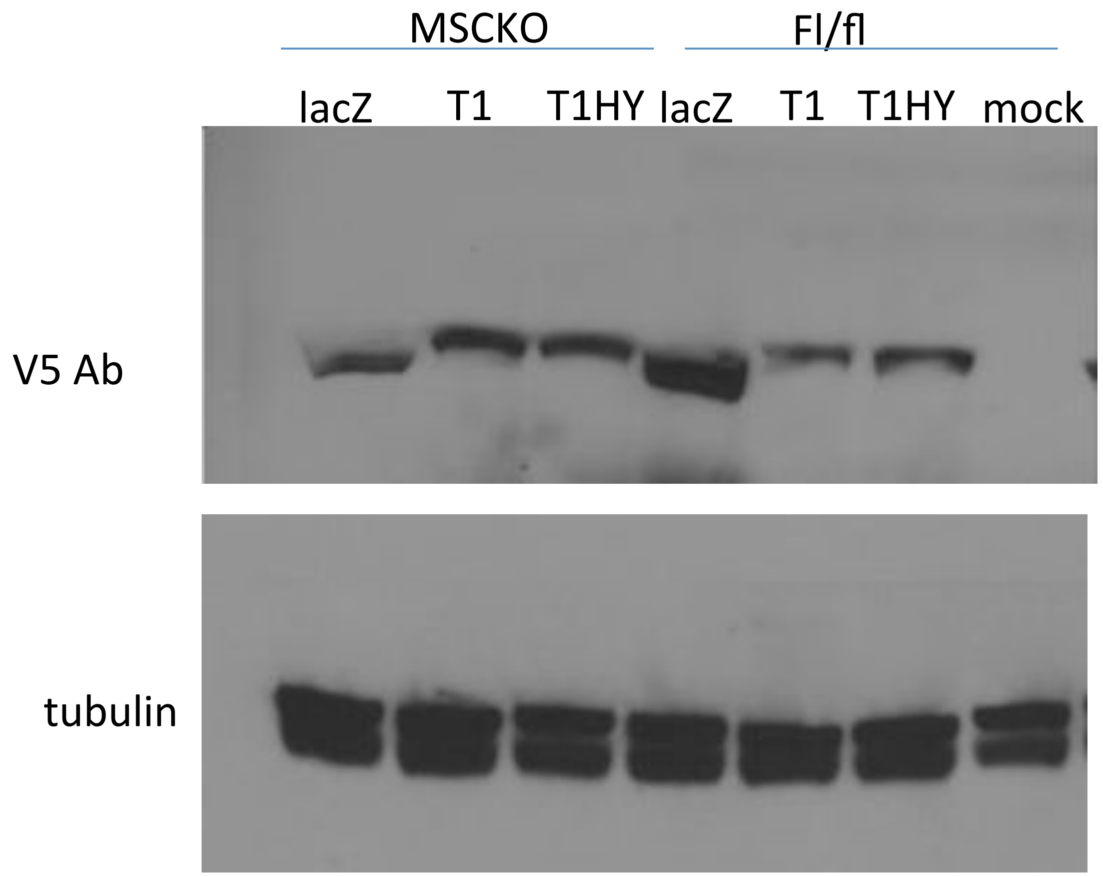

Supplementary Fig. 10.

A

geneset name	# genes in geneset (K)	# genes in overlap (k)	k/K	p value
STEMCELL_NEURAL_UP	1838	60	0.0326	1.07E-26
STEMCELL_EMBRYONIC_UP	1344	40	0.0298	1.86E-16
RUTELLA_HEMATOGFSNDCS_DIFF	661	29	0.0439	2.14E-16
BYSTRYKH_HSC_TRANS_GLOCUS	977	34	0.0348	4.64E-16
ALZHEIMERS_DISEASE_UP	1473	40	0.0272	3.80E-15
SIGNAL_TRANSDUCTION	1637	42	0.0257	4.94E-15
WNT SIGNALING – Β CATENIN TARGETS	122	7	0.057	1.9124E-06

B

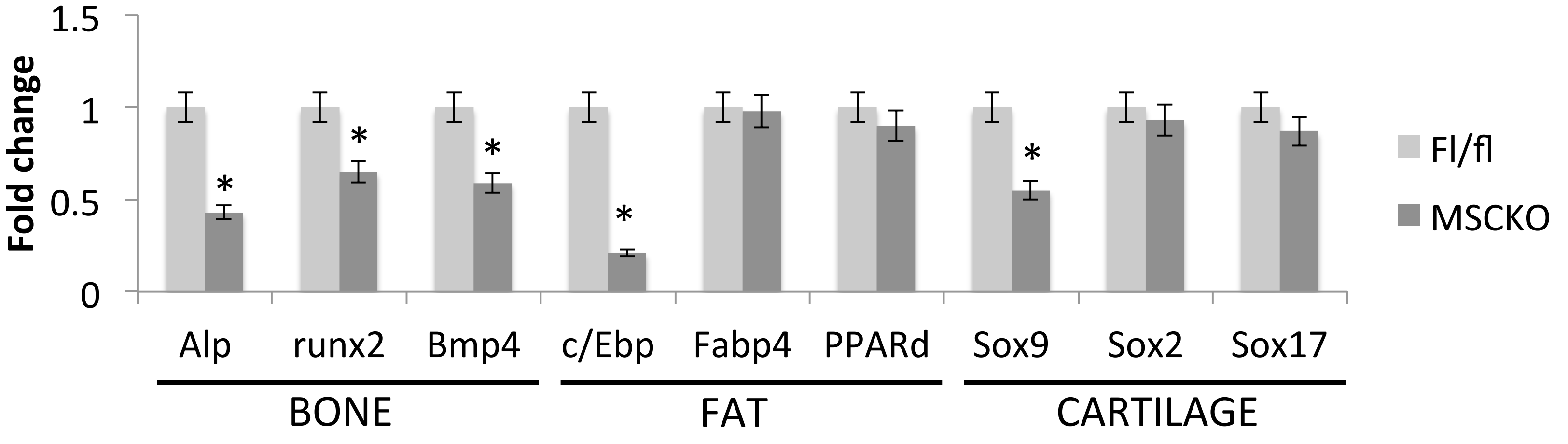

Supplementary Fig. 11.

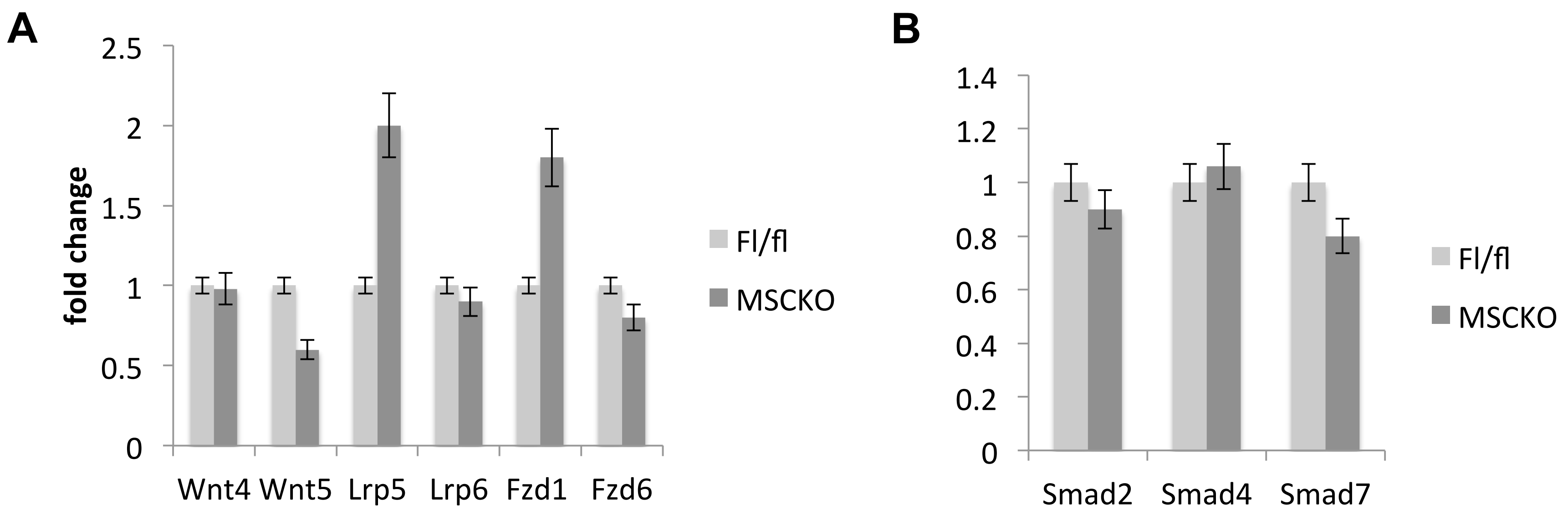

Supplementary Fig. 12.

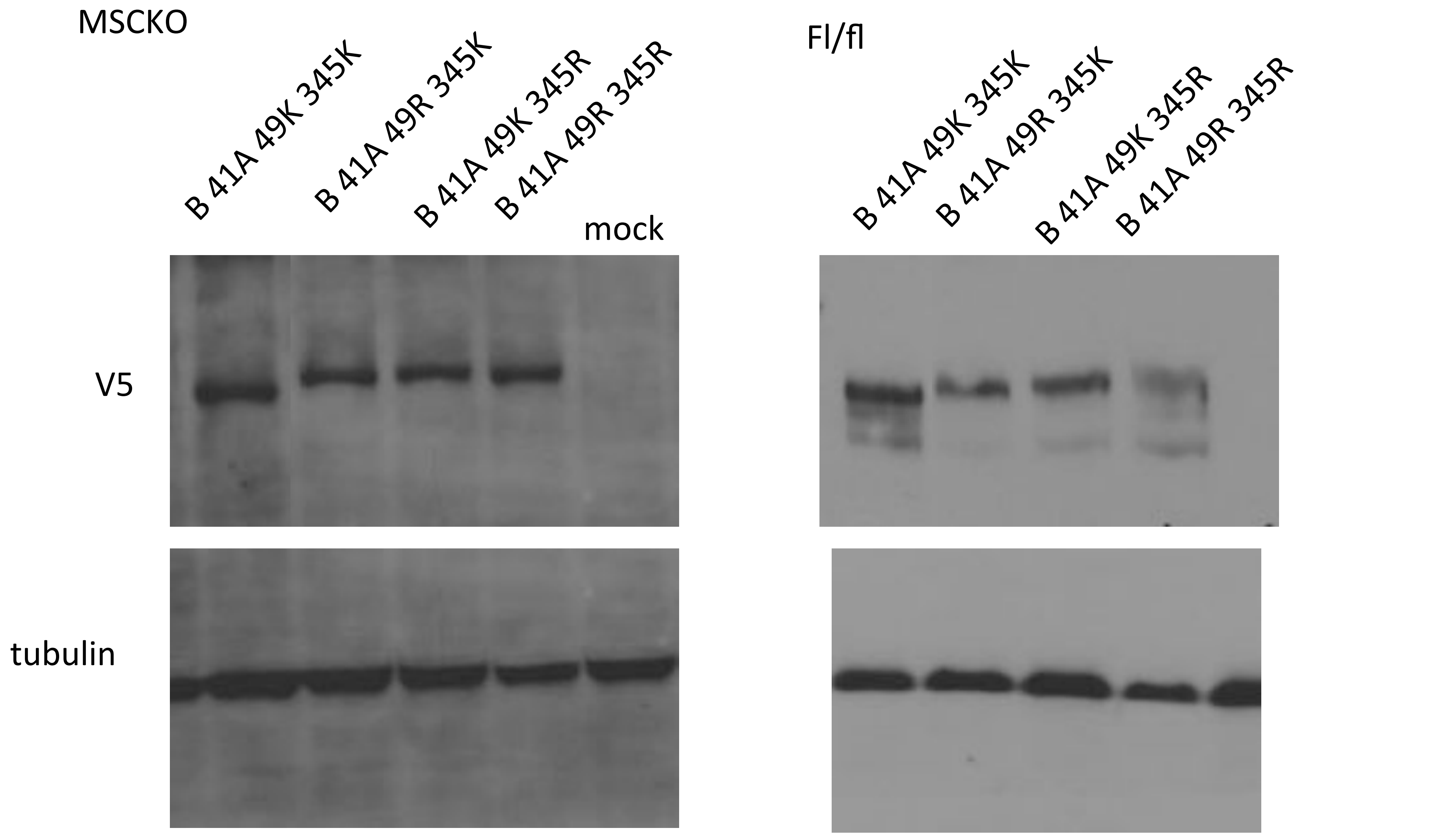

Supplementary Fig. 13.

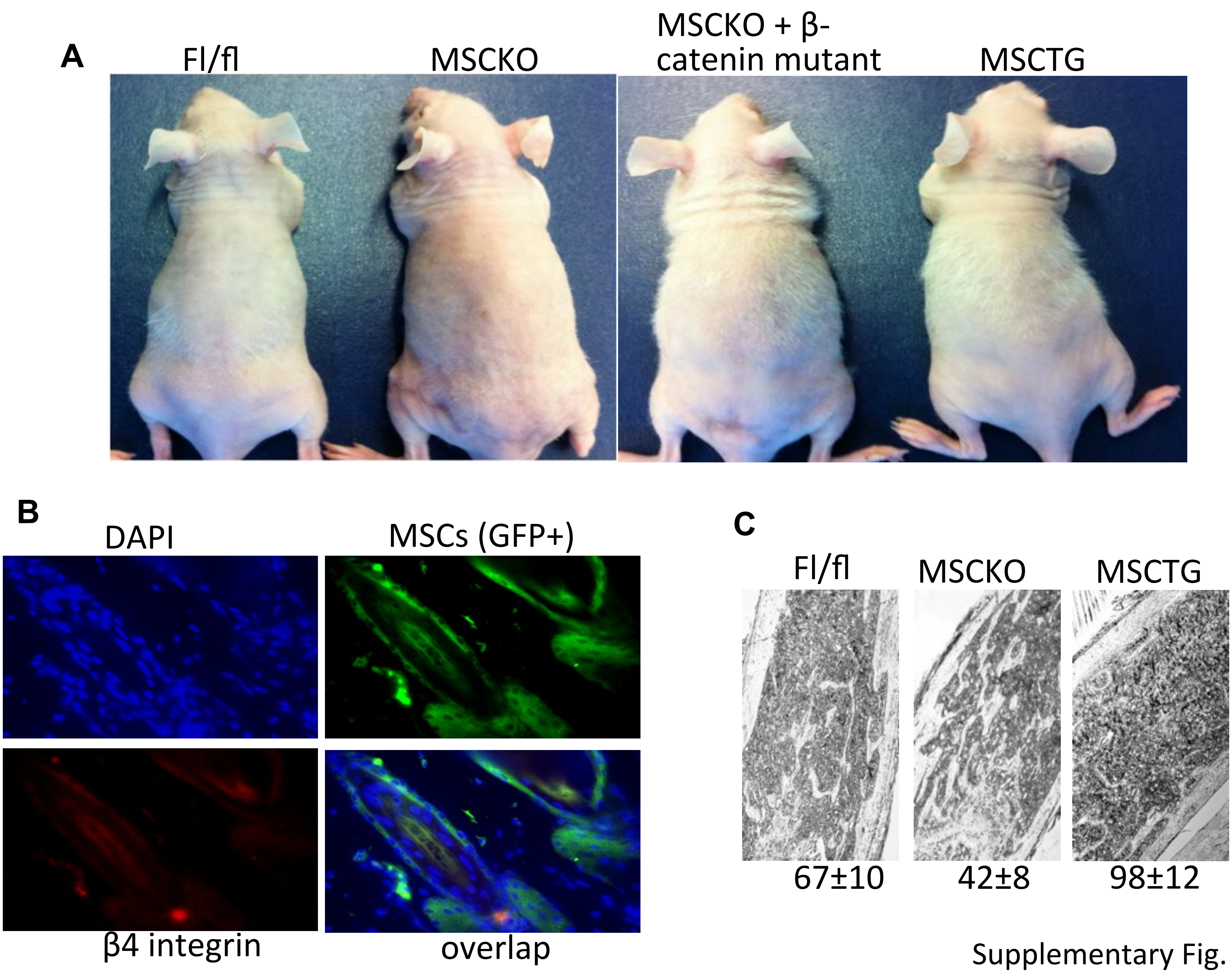

Supplementary Fig. 14.
